# Acquisition of Classifier Constructions in HKSL by Bimodal Bilingual Deaf Children of Hearing Parents

**DOI:** 10.3389/fpsyg.2018.01148

**Published:** 2018-07-23

**Authors:** Gladys W. L. Tang, Jia Li

**Affiliations:** Department of Linguistics and Modern Languages, Centre for Sign Linguistics and Deaf Studies, The Chinese University of Hong Kong, Shatin, Hong Kong

**Keywords:** bimodal bilingualism, word order, classifier constructions, language acquisition, HKSL, Cantonese, deaf children, coenrollment

## Abstract

The current study focuses on the acquisition of classifier constructions in Hong Kong Sign Language (HKSL) by a group of Deaf children of hearing parents, aided or implanted. These children have been mainstreamed together since kindergarten; but their learning environment supports dual language input in Cantonese and HKSL on a daily basis. Classifier constructions were chosen because previous research suggested full mastery at a late age when compared with other verb types, due to their morphosyntactic complexity. Also, crosslinguistic comparison between HKSL and Cantonese reveals differences in verb morphology as well as word order of the structures under investigation. We predicted that verb root and word order were the two domains for crosslingusitic interaction to occur. At the general level, given the specific learning environment and dual input condition, we examined if these Deaf child learners could ultimately acquire classifier constructions. Fifteen Deaf children divided into four groups based on duration of exposure to HKSL participated in the study. Two Deaf children born to Deaf parents and three native HKSL signers served as controls. A picture description task was designed to elicit classifier constructions containing either a transitive, a locative existential or a motion directional predicate. The findings revealed Deaf children's gradual convergence on the adult grammar despite late exposure to HKSL. Evidence of crosslinguistic influence on word order came from the Deaf children's initial adoption of a Cantonese structure for locative existential and motion directional predicates. There was also a prolonged period of adherence to the SVO order across all grades. However, within this SVO structure, the verb revealed increasing morphological complexity as a function of longer duration of exposure. We interpreted the findings using Language Synthesis, arguing that it was the selection of morphosyntactic features in Numeration that triggered crosslinguistic interaction between Cantonese and HKSL with bimodal bilinguals.

## Introduction

How deaf and hard-of-hearing children acquire language has always attracted attention among researchers in linguistics, speech and language pathology, and deaf education. In recent years, due to advancement in hearing technology, one also saw an increasing number of signing Deaf children demonstrating knowledge of spoken language either through print, or print and speech. To appreciate this development, one needs to understand the demography of Deaf children. Generally speaking, Deaf children who are born to Deaf parents (i.e., DDs) may acquire sign language since birth, and spoken language when they begin to receive speech training which usually comes as early as if not earlier than 1 year old. Hearing children who are born to Deaf parents (i.e., Kodas) usually acquire sign language and spoken language much earlier in life, if not simultaneously at birth. A great majority of Deaf children are born to hearing parents (i.e., DHs), and whose exposure to sign language depends largely on the type of formal education they receive. Some start to receive sign language exposure when their parents enroll them into schools for the deaf at various ages. Although integrative/inclusive education nowadays has led to a majority of DHs being mainstreamed without exposure to sign language, there is a small number of them whose education is facilitated by sign interpreters, hearing teachers who can sign and sometimes Deaf teachers. One such mode of bilingual education for the deaf in mainstream education is coenrollment, whereby a critical mass of deaf students study with hearing students in a mainstream classroom, supported by sign language and spoken language. This study focuses on this particular group of signing Deaf children whose parents enroll them into the Sign Bilingualism and Co-enrollment Education Programme in Hong Kong, generally referred to as the “SLCO Programme.” Through naturalistic interactions, these children receive Hong Kong Sign Language (HKSL) input consistently from 7 to 8 Deaf teachers and a critical mass of Deaf peers on a daily basis in the classroom/school setting (see section Participants), in addition to spoken language from their hearing teachers and peers.

Recently, researchers attempt to examine the bilingual acquisition of Kodas and DDs within the framework of *bimodal bilingualism*, defined as acquisition and use of a sign language and a spoken language that stem from the visual-gestural and the auditory-oral modalities respectively. From a child language perspective, bimodal bilingualism has been associated with bilingual first language acquisition, which, in the spoken language literature, is further categorized into *simultaneous* and *sequential bilingual acquisition*. The former refers to acquisition of two languages at the same time since birth while the latter requires exposure to a second language at a very young age and usually no later than 5 (see Meisel, 2011). A general characteristic of bimodal bilingual acquisition is *code blending*, defined as simultaneous and systematic production of sign and speech^1^. A number of studies targeting Kodas and DDs reveal a prevalence of congruent code blends and the challenge is how to account for the incongruent ones (Petitto et al., 2001; van den Bogaerde and Baker, 2005; Lillo-Martin et al., 2010; Donati and Branchini, 2013; Fung and Tang, 2017). Additionally, in the spoken language literature, while it is generally agreed that bilingual children separate the two grammars from earlier on, systematic crosslinguistic influence is also at play. Hulk and Müller (2000) argue for two conditions for crosslinguistic influence to occur, namely interface between pragmatics and syntax, and structural overlap at the surface level. These two conditions have been subject to investigation in many bilingual acquisition studies. As for bimodal bilingualism, research shows that crosslinguistic influence is observed in structures not predicted by such conditions (Lillo-Martin et al., 2010), and findings for the structures that satisfy these two conditions run counter to predictions (Koulidobrova, 2012, 2016). Recently, Language Synthesis (Koulidobrova, 2012, 2016; Lillo-Martin et al., 2012, 2014, 2016) has been proposed to account for the various language interaction effects observed in bimodal bilingualism (see the next section). The proposal is based on MacSwan's (2000, 2005) accounts for code-switching, in which he argues for one computational system with separate lexicons and separate Phonetic Forms (PFs) for different languages.

This study focuses on another language pair, HKSL and Cantonese, and investigates how Deaf bimodal bilinguals born to hearing parents acquire classifier constructions in HKSL. This structure was chosen because full mastery has been reported late due to its morphosyntactic complexity [ASL (American Sign Language): Supalla, 1982; Schick, 1990; HKSL: Lam, 2009]. Additionally, while sharing SVO as the basic word order, HKSL and Cantonese differ in verb morphology. Cantonese is said to be poor in inflection; verbs are bare and the basic word order is consistently SVO. On the contrary, HKSL is rich in inflection and the morphsyntactic properties of the verb interact with word order changes. These crosslinguistic differences invite an examination of how bimodal bilingual Deaf children develop knowledge of verb morphology and word order in classifier constructions in HKSL. Additionally, we also explore if DHs can acquire knowledge of such complex constructions as a function of duration of exposure, given the fact that they fail to receive early HKSL input since birth. Last, we examine to what extent Language Synthesis may account for the language interaction effects observed in this study. Evidence supporting Language Synthesis is rather limited, hence further exploration to identify the conditions for language interaction effects to occur is necessary.

The paper is organized as follows. We will first summarize the word order issues that have been documented in bimodal bilingual acquisition of a number of language pairs. Then, we introduce Language Synthesis, recently proposed to account for language interaction effects such as code blending, code switching, as well as crosslingusitic influence and transfer (Lillo-Martin et al., 2016). Based on these discussions, we compare the verb root and word order issues with the relevant constructions between Cantonese and HKSL. We then set out some predictions about how crosslinguistic interaction may occur. The experimental procedure, backgrounds of the DHs and the results are then summarized and discussed. At the end of the paper, we will discuss some caveats of the study and offer suggestions for future research.

## Background

### Previous acquisition research on word order in sign languages

In the early literature on ASL acquisition, canonical SVO and derived word orders are observed to emerge at an early age among DDs (Newport and Meier, 1985; Lillo-Martin, 1999; Chen Pichler, 2001). However, Lillo-Martin and Berk (2003) found that the two DHs in their study, who were not exposed to an accessible language like ASL until after age 5, had no problem acquiring the canonical SVO order but seldom attempted derived word orders that reflected grammatical dependencies and erred more when they did so. Reports involving bimodal bilinguals especially Kodas are emerging in recent years. Based on the longitudinal data (ages ranged from 2;00 to 4;00) of two ASL-English and one Brazilian Sign Language (Libras)-Brazilian Portuguese (BP) Kodas, Lillo-Martin et al. (2010:272) observed doubling in the English data (e.g., “sleeping mouse sleeping,” Ben 2;01). Putting forward *Language Synthesis* as an overarching framework of analysis, they argued that the doubling phenomenon may be captured by the choice of a functional element with a [+focus] feature from ASL in the Numeration and late insertion of lexical items in English. In another study, the same team of researchers examined the same Kodas' (age 1;11–4;05) production of *wh*-questions (Lillo-Martin et al., 2012). According to them, English and BP allow fronted and *in-situ wh*-questions only whereas ASL and Libras's *wh*-questions allow more syntactic options: fronted, *in-situ*, and doubled (e.g., ASL: WHO JOHN SEE WHO “Who did John see?”). Generally speaking, they observed emergence of *in-situ wh*-questions earlier with bimodal bilinguals than monolinguals of either spoken language. Additionally, while monolingual English and BP child acquirers produced fronted *wh*-questions exclusively, bimodal bilinguals' *wh*-questions in English were fronted, *in-situ* as well as doubled. What is also interesting is that these doubled *wh*-questions began to retreat from the English of the two Kodas after 2;11. Using an elicited production task on a larger sample of Kodas, the researchers found a much higher rate of production of *wh*-initial questions in ASL by the Kodas than the Deaf controls. Recently, Palmer (2015) compared the acquisition of ASL canonical and non-canonical word orders of four bimodal bilinguals, two Kodas and two implanted DDs whose ages ranged from 1;8 to 3;6. While both the Kodas and DDs produced canonical SV and VO orders as early as 23 months, suggesting an early setting of Spec-Head and Head-Complement parameters, they showed little use of non-canonical OV and VS orders when compared with the Deaf controls as reported in Chen Pichler (2001).

In the HKSL context, few acquisition studies focus on the relation between word order and verb root of classifier constructions. Tang et al. (2007) elicited simultaneous constructions from a group of DHs who studied in a school for the deaf (ages ranged from 6 to 13). They used comic strips to elicit narratives from these participants. The DHs did not introduce the antecedent before the classifier predicate, nor did they sustain the classifier on the non-dominant hand in space that refers to the direct or indirect object. Lam (2009) in a longitudinal study of a DD acquiring HKSL found that the nominal antecedent is usually not overtly expressed but recoverable from the signing discourse. Although the first token of OV order with a classifier predicate involving one argument emerged at age 2;9.29, very few OV or SOV orders were observed throughout. Instead, the Deaf child produced primarily VO (46.67%) and SVO (33.33%) orders with a classifier predicate during the observation period, which we interpret to be illicit word orders for this structure. Lam (2009) ascribed it to the optionality of object shift that delayed full acquisition. Indeed, one needs to address why the Deaf child accepted an (S)VO order for classifier constructions. We predict that language interaction effect associated with the canonical SVO order of Cantonese and HKSL might be the cause of this acquisition phenomenon (see section Crosslinguistic Comparison and Acquisition Predictions).

### Emerging accounts for language interaction effects

As previously discussed, Language Synthesis has been put forward to account for code switching, code blending, crosslinguistic influence in early language development, transfer in second language acquisition, and calquing in language contact situations (see Lillo-Martin et al., 2016 for a detailed illustration). This model has its basis in Distributed Morphology, which posits that it is the selected roots and atomic features in the Numeration (i.e., List 1) that enter the syntactic computation, and insertion of Vocabulary Items from List 2 is a late phenomenon taking place after Spell-Out to the PF branch (Harley, 2014). According to Language Synthesis, List 1 and List 2 are the two places at which interaction between Lx and Ly may occur. When the atomic features are selected from Lx but Vocabulary Insertion draws items from Ly, syntactic synthesis (i.e., crosslinguistic influence, transfer, and calquing) results. Embracing two paths toward PF after Spell-out, one for sign and the other for speech, the process allows simultaneous realization of the possible mix of elements from Lx and Ly, resulting in code blending. Additionally, Lillo-Martin et al. (2016) suggest that the apparent crosslinguistic influence is actually bimodal bilingual effects, meaning that in the constant absence of forced language choice (i.e., inhibition), bimodal bilinguals are “accustomed” to practicing choosing grammatical elements between Lx and Ly during Numeration and Vocabulary Insertion. The so-called crosslinguistic influence is only a reflection of bimodal bilinguals' capacity for language synthesis.

Language Synthesis has attracted a lot of debates about its explanatory adequacy, particularly, for cases where bimodal bilinguals produce two independent strings in diverse word orders simultaneously, as in the code blending between Italian/LIS (Italian Sign Language) or Dutch/NGT (Sign Language of the Netherlands) that involves divergent SVO vs. SOV orders (Baker, 2016; Branchini and Donati, 2016), as the example in (1) shows:

(1)
  **Table:** 

Italian:	Non	ho	capito
	not	have.1SG	understand. PTCP
		__NEG	
LIS:	UNDERSTAND	NOT	
	“I don't understand.”

          (Branchini and Donati, 2016, example 13).

Instead of one mixed Numeration and late Vocabulary Insertion as what Language Synthesis suggests, Branchini and Donati (2016) argue that bimodal bilinguals have at their disposal two separated monolingual Numerations and two parallel syntactic derivations. They have identified three types of code blendings. The first type (Type 1) has one syntactic representation the derivation of which is based on one Numeration and governed by a single grammar of either LIS or Italian. The output displays all the necessary properties of the language dictating the representation. As bimodal bilinguals are equipped with a double spell-out, lexical retrieval from the “governed” language to derive code blending or fragment insertion in code switching can take place at a late stage, hence it will not affect the grammatical representation of the “dictating” language. As a result, the governed language is impoverished in terms of morphological and phonological properties. The second type (Type 2) involves two strings with independent representations and full-fledged morphological and phonological properties, as in (1) above. This type is often observed when two languages have a rigid word order for functional elements (e.g., the position of negators in Italian is preverbal while in LIS it is postverbal). They argued that such occurrences are due to two parallel Numerations and syntactic derivations. The third type (Type 3) like (2) below have two simultaneous strings that “contribute together to form a unique utterance” (Branchini and Donati, 2016 p.21). Type 3 differs from Type 1 in that both language strings are not impoverished in any sense; it also differs from Type 2 in having one mixed, not two separated Numeration which contributes to a single derivation. In example (2), the subject (i.e., I) is provided by Italian while the predicate (i.e., WIN) by LIS. Only when both language strings are taken into account together will the utterance become complete and meaningful. Based on grammaticality judgment and elicited production data, Branchini and Donati confirmed that all three types are part of the Kodas as well as the adults' grammar, hence not developmental. Additionally, Type 3 is akin to what Language Synthesis stipulates, where merging roots and morphemes from two different languages is possible in the Numeration initially.

(2)
  **Table:** 

Italian:	io
	1SG
LIS:	WIN
	“I win.”

(Branchini and Donati, 2016, example 37).

Both Language Synthesis and the proposal by Branchini and Donati (2016) share the assumption that bimodal bilinguals are characterized by co-activation and non-inhibition during bilingual processing. They diverge in the theoretical assumptions about (a) whether there is a list of morphosyntactic features or a Lexicon to store lexical items with pre-assembled features; and (b) whether there is only one mixed Numeration to drive a single derivation or two separate Numerations to drive two parallel syntactic derivations. While these proposals are originally developed to account for bilingual first language acquisition of Kodas, it is possible to extend the analyses to examine language interaction effects in the developing grammars of bimodal bilingual Deaf children from hearing families. So far, the Language Synthesis model has been adopted to account for word order data. The current study aims to extend the analysis to the interaction between word order and morphosyntactic features as involved in classifier constructions. Additionally, we adopt Distributed Morphology in our analysis of classifier constructions in HKSL because we assume it is a “list” of morphosyntactic features, not a Lexicon, that forms the basis for Numeration. However, we are open to Branchini and Donati's (2016) proposal for the possibility of two independent Numerations.

### Word order

#### Verb morphology and word order in HKSL

Similar to other sign languages, word order in HKSL interacts with verb morphology. Verbs in HKSL can be categorized into three types, i.e., plain verbs, agreement verbs, and spatial verbs. Plain verbs such as LIKE and THINK are generally without inflectional morphology; spatial verbs like PUT and TAKE can be modified through movement to the R-loci of location arguments, and agreement verbs like HELP, PUSH, and GIVE associate the R-loci with the subject and/or (indirect) object in terms of person and number. According to Sze (2000), the canonical word order in HKSL is SVO with plain verbs (3a,b) and SOV with agreeing and spatial verbs (3c,d)^2^.

(3a)
  **Table:** 

HKSL:	FATHER	LIKE	BOY
	“Daddy likes boys.”

(3b)
  **Table:** 

^*^HKSL:	FATHER	BOY	LIKE
	“Daddy likes boys.”

(3c)
  **Table:** 

HKSL:	LAST^∧^NIGHT	FATHER_a_	POLICEMAN_b_	_a_HELP_b_
	“Last night, father helped the policeman.”

(3d)
  **Table:** 

HKSL:	LAST^∧^NIGHT	FATHER	BOOK_i_	PUT_a−i_
	“Last night, father put the book (there).”

Note that agreement markings may be optional in HKSL; therefore, the order becomes SVO rather than SOV with uninflected agreement verbs (4a,b).

(4a)
  **Table:** 

HKSL:	FATHER	HELP	BOY	IX_a_
	“Daddy helps the boys there.”

(4b)
  **Table:** 

^*^HKSL:	FATHER	BOY	IX_a_	HELP
	“Daddy helps the boys there.”

#### Analysis of classifier constructions in HKSL

There has been much debate about the grammatical status of classifier constructions. The iconic and mimetic nature of object and event depiction in classifier constructions has resulted in claims by some researchers that the term “classifier” is a misnomer. Instead, alternative terminologies have been suggested, such as “visual schematic representations” (Cogill-Koez, 2000), “depicting verbs/constructions” (Cormier et al., 2012), or “polycomponential verbs” (Schembrei, 2003). Nonetheless, there are attempts to adopt a morphosyntactic analysis of classifier predicates in different sign languages. Supalla (1982, 1986) analyzing ASL proposes that classifier predicates are composed of movement roots and a set of affixes, among which handshapes and locations are obligatorily affixed to the verb stem and function as agreement markers. Within the framework of Minimalism, Benedicto and Brentari (2004) argue for the role of classifiers as mophosyntactic markers for external and internal arguments in transitive-intransitive and unergative-unaccusative alternations. They also posit that classifiers are heads of functional projections, i.e., *f*_1_P or *f*_2_P, with morphosyntactic features which agree with those of an argument in the specifier position (i.e., structural agreement). Therefore, movement of an argument selected by the VP is either to an external argument position (i.e., Spec, *f*_1_P) or an internal argument position (i.e., Spec, *f*_2_P). However, unresolved issues remain, such as how body part classifiers and instrumental classifiers fit into the picture.

An alternative agreement analysis based on Distributed Morphology for classifier predicates is put forward by Glück and Pfau (1998, 1999), who argue that both agreement verbs and classifying verbs share a similar morphological paradigm of agreement, in terms of moving between R-loci to show subject/object-verb agreement. But for classifying verbs there is another type of agreement, which is agreement between handshapes and the arguments they are denoting. This similarity is taken up in Zwitserlood (2003, 2008) who argues that classifiers have features for handshape and locus to spell out agreement in the structure^3^. At Numeration, the associated morphosyntactic feature bundles as well as a verb root are selected from List 1 and merged to form “root phrases” (rootPs) until a categorical “little *v*P,” a cyclic domain boundary for Spell-Out, is formed. This structure is shipped off to LF (Logic Form) for semantic interpretation and to PF for Vocabulary Insertion. At this stage, morphological operations apply on the PF branch, which is merger of agreement nodes for classifiers and R-loci, altering the syntactic structure hence word order changes accordingly. Vocabulary Items (i.e., elements from List 2) then compete for phonological realizations of the terminal nodes emerging from the syntactic structure. On the LF branch, the conceptual/intentional interface looks for interpretations for each terminal node (i.e., elements of List 3).

In this study, we will adopt the agreement analysis to account for the classifier constructions in HKSL and the related acquisition phenomenon. Following Zwitserlood (2003, 2008) and Glück and Pfau (1998, 1999), we assume there is agreement based on the handshape features and the antecedents; and at the same time, subject and object agreement can be spelt out through movement of the handshape classifiers between loci in space. At the descriptive level, classifier constructions in HKSL generally follow the schema of introducing the Ground before the Figure, as shown in (5a–5c)^4^. In other words, the Ground, like the theme NP in (5a) (i.e., BACKPACK), locative NP in (5b) (i.e., TOILET^∧^ROLL), and goal NP in (5c) (i.e., TOY^∧^CAR), is introduced into the discourse first through a locative predicate, with a classifier on the non-dominant hand being assigned to an R-locus in space. This classifier is sustained in space when the dominant hand introduces the Figure and a second classifier predicate, a phenomenon referred to as “perseveration.” Note that in accounting for spatial expressions in NGT, Pfau and Aboh (2012) claim that the Part of the Ground (e.g., top of/next to the house) as expressed by H2 is usually left unexpressed. However, according to the native Deaf signers of HKSL, it is usually overt, consistently displaying a two-handed simultaneous, hence a Figure-Ground construction for the transitive predicate (5a), the locative existential predicate (5b), and the motion directional predicate (5c). Sometimes, the introduction of the object is simply by a topic (6a), or the object following the subject in an SOV order (6b).

(5a)
  **Table:** 

HKSL:	DH:	BACKPACK_i_		CAT_j_	_b_push_a_+CL_SEMj_
	NDH:		be_located_a_+CL_SASSi_	————————>—————————–	
		“The backpack is located here; the cat pushes it (with its side).”

(5b)
  **Table:** 

HKSL:	DH:	TOILET^∧^ROLL_i_		SCISSORS_j_	be_located_on_a_+ CL_SASSj_
	NDH:		be_located_a_+CL_SASSi_	————————–>———————————	
		“The toilet roll is located here; the pair of scissors is located (on) it.”

(5c)
  **Table:** 

HKSL:	DH:	TOY^∧^CAR_i_		DOG_j_	_b_jump_onto_a_+CL_SEMj_
	NDH:		be_located_a_+CL_SEMi_	————————>—————————–	
		“The toy car is located here; the dog jumps onto it.”

(6a)
  **Table:** 

		__________top			
HKSL:	DH:	IX_a_	DOOR_i_		_a_open_b_+CL_HANDj_
	NDH:			MOTHER_j_	be_located_a_+CL_SASSi_
		“That door, mother opens it.”

(6b)
  **Table:** 

HKSL:	DH:	BOY_i_	VASE_j_	_b_kick_a_+CL_BODYPARTi_
	NDH:			be_located_a_+CL_SASSj_
		“The boy kicks the vase with his leg.”

Following Distributed Morphology and Zwitserlood (2003), we assume the root of a classifier predicate merges with different arguments bearing bundles of features to form rootPs, and eventually reaches a category node little *v*P, at which point the structure is shipped off for Spell-Out. At PF, the movement specification for the verb is inserted at the terminal node and different agreement projections are further merged above little *v*P. Subsequently, the feature bundles at Agr nodes, including the respective handshape and locus features for the Figure and Ground, are spelt out as classifier and spatial agreement markers via subject and object agreement respectively. Note that the arguments that are merged with the verb root vary in accordance with the predicate types. For (5a), the locative existential predicate “be_located” requires a Theme and a Location argument and projects an AgrS and AgrIO nodes above little *v*P. For (5b), the motion directional predicate “jump” requires arguments for Theme, Source, and Goal and projects an AgrS and two AgrOO (oblique object) nodes. Finally, the transitive predicate “push” in (5c) requires an Agent argument for AgrS and a Theme argument for AgrDO. Basically, all the AgrS nodes will be spelt out and inserted with the phonological specification for classifiers of the Figure. This includes the external argument of unergative and transitives as well as the internal argument of unaccusative predicates at the specifier of AgrS. For (5a–c), the classifier presenting the Ground argument, which refers to the object in an OSV order, is localized at an R-locus with which the movement of the Figure argument has to “agree” both in terms of spatial and grammatical agreement^5^. The phenomenon of perseveration shows that the classifier on H2 is an anaphoric expression which co-refers to the Ground argument introduced initially into the signing discourse. Although in the discourse the locative predicate following the Ground is omitted sometimes, the perseveration of the classifier on H2 at an R-locus is still observed in the predicate, like (6a). Therefore, we assume that the classifier on H2 inside the simultaneously articulated predicate is merged at the Spec position of the object agreement nodes and the syntactic derivation follows. The Figure may undergo movement to a functional projection higher than the Ground, to form the less frequently used SOV order like (6b).

To sum up, SVO order is not allowed in classifier constructions containing two noun referents in HKSL, while OSV based on a Ground-Figure schema is more frequently used than SOV. The descriptions above offer a framework to elucidate the syntactic function of moved or *in-situ* subjects and objects, as well as the status of classifiers as functional elements whose morphosyntactic features agree with the noun referents.

#### Cantonese counterparts of classifier constructions

Cantonese, though a classifier language, differs from HKSL in having numeral classifiers in the nominal as well as verbal domains. In (7a), *go3* is a nominal classifier and *kyun4* a verbal classifier. Also, verbs in Cantonese lack overt morphological agreement marking and grammatical relations are expressed primarily through the SVO order, as shown in the transitive (7a,b), locative existential (7c,d) and motion directional predicates (7e).

(7a)
  **Table:** 

Cantonese:	**go3**	naam4zai2	daa2	zo2	**go3**	tung4hok4	saam1	**kyun4**
	CL	boy	hit	PERF	CL	schoolmate	three	CL
	“The boy punched the schoolmate three times with his fist.”

(7b)
  **Table:** 

Cantonese:	**zek3**	maau1	teoi1	gan2	**go3**	syu1baau1
	CL	cat	push	PROG	CL	school-bag
	“The cat is pushing the school bag.”

(7c)
  **Table:** 

Cantonese:	**jau5**	baa2	gaau3zin2	**hai2**	gau6	ci3zi2	**soeng6min6**
	have	CL	scissors	be located	CL	toilet roll	(on the) top of
	“A pair of scissors is on the top of the toilet roll.”

(7d)
  **Table:** 

Cantonese:	gyun2	ci3zi2	**soeng6min6**	**jau5**	baa2	gaau3zin2
	CL	toilet roll	(on the) top of	have	CL	scissors
	“A pair of scissors is on the top of the toilet roll.”

(7e)
  **Table:** 

Cantonese:	zek3	gau2	tiu3 soeng6heoi3	gaa3	wun6geoi6ce1	**soeng6min6**
	CL	dog	jump up.go	CL	toy car	(on the) top of
	“The dog jumps onto the top of the toy car.”

There are two alternative constructions for locative existentials in Cantonese. While maintaining an SVO order, (7c) uses a locative verb *hai2* “be located” and (7d) an existential verb *jau5* “have.” Additionally, the locative NP is marked by a localizer^6^ *soeng6min6* (on top of). Note that in the literature, a clause initial *jau5* “have” is analyzed as an existential quantifier introducing an indefinite NP *baa2 gaau3zin2* “a pair of scissors” into the discourse, as in (7c). In (7d), *jau5* “have” is analyzed as an existential verb selecting a locative NP as the grammatical subject (Huang, 1990). As for motion directional predicates, an SVO order maintains but the verbal domain is composed of serial verbs *tiu3 soeng6heoi3* “jump onto” in (7e).

### Crosslinguistic comparison and acquisition predictions

The grammatical descriptions above show crosslinguistic differences between HKSL and Cantonese regarding the three types of constructions (i.e., transitive, locative existential, and motion directional constructions), both in terms of word order and morphological complexity of the verb root. Classifier constructions in HKSL are primarily OSV, and sometimes SOV, while the equivalent constructions in Cantonese are consistently SVO. Second, following Distributed Morphology, for the selection of morphosyntactic features from List 1, HKSL differs from Cantonese in the selection of roots, classifier features and locus features to mark subject/object as well as spatial agreement at the R-loci of the classifiers. As said, the selection of locus feature in the Numeration is crucial for spatial agreement function as they spell out the R-loci for the classifiers in space. Such properties are absent in Cantonese. Furthermore, locative existentials in Cantonese are explicitly encoded by a locative verb *hai2* “be located” or an existential verb *jau5* “have” and a localizer like *up*, whereas in HKSL such constructions require an abstract verb root *be_located* and some placement affixes such as “next to” and “on top of” to encode the axial parts of the Ground entity with which the Figure sets up a spatial relation with. These crosslinguistic differences between HKSL and Cantonese pose interesting acquisition predictions especially in the context of Deaf children acquiring HKSL in a bilingual fashion.

As discussed previously, the basic word order of HKSL is SVO with plain verbs, uninflected spatial and agreement verbs. Child data from Lam (2009) also confirmed an initial SVO order based on plain verbs. As such, it overlaps with the canonical SVO order in Cantonese. Under these circumstances, we predict that the initial word order of constructions involving a classifier predicate in HKSL is SVO, which may actually be doubly enhanced by the “shared” canonical SVO order of Cantonese and HKSL. Language Synthesis will predict that these DHs may initially select those morphosyntactic features pertaining to a SVO order with a lexicalized verb root, but not classifier features or locus features. Under those circumstances, it pertains to a Cantonese or a HKSL-based structure and the latter reflects the word order grammar of plain verbs and sometimes uninflected agreement verbs. As such, Vocabulary Insertion may come from Cantonese and HKSL, or both under code blending conditions.

Subsequent acquisition of inflectional morphology for person and spatial agreement with agreement verbs and spatial verbs may trigger Deaf children's reanalysis of verb morphology, in the sense that HKSL verbs are not totally uninflected, leading to a reformulation of sub-classes of verbs and one of them is classifier constructions constituted by an abstract verb root, classifier features as well as locus features for spatial and subject/object agreement. We predict that classifier features are selected earlier than locus features in the Numeration, because classifier features, said to be akin to gender features in Zwitserlood (2003), are more semantic in nature, unlike locus features which yield R-loci in space for certain formal functions of encoding referential and agreement relations. The selection of such features in the Numeration motivates projections of agreement nodes at Spell-out where the features are merged at the terminal nodes for Spec-Head agreement with the noun referents in the specifier positions, and for spelling out the R-loci of the classifiers for subject/object agreement. In other words, the acquisition of the morphosyntactic properties of classifier constructions, and the schema of the Ground preceding the Figure in classifier constructions trigger Deaf children to develop word order variation, from SVO to OSV or SOV orders.

To sum up this section, we examine whether the selection of morphosyntactic features in the Numeration is a potential domain for language interaction to occur in our DHs' production of HKSL classifier constructions. Lack of inhibition also implies that Vocabulary Insertion as a late phenomenon allows items to come from either Cantonese or HKSL.

## Methodology

### Participants

The current study involved 15 HKSL-Cantonese DHs who have been mainstreamed into a sign bilingual and co-enrollment (SLCO) environment in Hong Kong since kindergarten. The SLCO classes, comprised of Deaf and hearing students in a ratio of 1:3 or 1:4, are co-taught by a hearing teacher and a Deaf teacher who is either a native or a near-native signer of HKSL. Totally, there are about 7 to 8 Deaf teachers in school who use primarily HKSL as the language of instruction and communication with other teachers and students, Deaf and hearing. The hearing teachers use primarily Cantonese and English, and sometimes Mandarin Chinese as the language of instruction; however, they also sign to facilitate communication whenever necessary. As both Deaf and hearing children are bimodal bilingual, they usually switch between Cantonese and HKSL in their daily interactions. At the time of the experiment, the DHs came from Primary 3 to Primary 6. Being DHs, the school is the only learning environment in which they receive consistent input in HKSL, in addition to Cantonese at home and at school. Note that they had HKSL exposure 1 h per week for 8–12 months before joining the SLCO Programme. In this study, we took the age of acquisition (AoA) of HKSL at the point when they started to receive consistent and ample input in HKSL in the SLCO Programme. At the time of the experiment, their chronological ages ranged from 8;10 to 14;5. Their AoA of HKSL ranged from 4;2 to 7;2. For five of these students, they could also be considered as late learners of HKSL due to exposure to the language at roughly age 6 or 7^7^.

We divided these 15 DHs into four groups on the basis of their duration of exposure to HKSL. Each group differed from the others by 1 year of exposure to HKSL. The DHs in Group 1 (aver. AoA of HKSL = 73.5 months) had the longest duration of exposure to HKSL for about 7 years. Those in Group 2 (aver. AoA of HKSL = 68.25 months), Group 3 (aver. AoA of HKSL = 55 months), and Group 4 (aver. AoA of HKSL = 59 months) had around 6, 5, and 4 years of exposure to HKSL, respectively. The numbers of DHs in each group were 4, 4, 4, and 3 for Groups 1, 2, 3, and 4, respectively. Among all of the 15 DHs, 11 of them have profound hearing loss (91+ dB), 3 of them are severely deaf (71–90 dB), and 1 have moderately severe hearing loss (56–70 dB). All of the 11 profoundly DHs are implanted, excluding 3 of them who wear hearing aids. Except for hearing loss, all of them do not have any other disabilities.

Two Deaf children of Deaf parents (DD-1 and DD-2), who are siblings to each other, took part in the current study as controls. DD-1 (studying with students of Group 1) is 1 year older than DD-2 (studying with children of Group 2). Due to misconception about sign language in HK earlier on, these two DDs did not have intensive HKSL exposure until 1;9 and 1;3 respectively; however, we suspect casual viewing of HKSL occurred at home since both of their parents are Deaf. DD-1 and DD-2 have been studying in the same SLCO Programme as the other 15 DHs. Their chronological ages were 12;9 and 11;3 respectively at the time of the experiment. Table 1 summarizes the background information of the 15 DHs and 2 DDs.

**Table 1:** Backgrounds of DHs and DDs.

**Participants**	**Research Code**	**Gender**	**Deaf parent(s)**	**Grade**	**Age (month)**	**HKSL AoA (month)**	**Duration of HKSL exposure (month)**	**Degree of hearing loss in the better ear (dB)**	**Hearing device**	**Age of wearing CI/HA (month)**
Group 1	DH-G1-1	F	No	P6	140	52	88	88	CI	27
	DH-G1-2	F	No	P6	167	80	87	118	CI	41
	DH-G1-3	M	No	P6	163	76	87	105	CI	38
	DH-G1-4	M	No	P6	173	86	87	108	CI	71
Group 2	DH-G2-1	M	No	P5	129	54	75	108	CI	30
	DH-G2-2	M	No	P5	154	79	75	107	HA	38
	DH-G2-3	M	No	P5	150	66	84	87	HA	36
	DH-G2-4	F	No	P5	149	74	75	120	HA	3
Group 3	DH-G3-1	F	No	P4	122	54	68	93	HA	26
	DH-G3-2	F	No	P4	127	58	69	97	CI	23
	DH-G3-3	M	No	P4	127	58	69	60	HA	33
	DH-G3-4	F	No	P4	120	50	70	108	CI	24
Group 4	DH-G4-1	F	No	P3	119	63	56	120	CI	22
	DH-G4-2	F	No	P3	119	63	56	120	CI	22
	DH-G4-3	M	No	P3	106	51	55	85	HA	43
DD	DD-1	M	Yes	P6	153	21	132	93	HA	6
	DD-2	F	Yes	P5	135	15	120	72	HA	31

For a better understanding of their knowledge of spoken languages, Cantonese and written Chinese assessments are administered to the 15 DHs and 2 DDs annually, which are the *Assessment of Chinese Grammatical Knowledge (ACGK)*, and the subscale on *Cantonese Grammar of Hong Kong Cantonese Oral Language Assessment Scale (HKCOLAS-CG)* (T'sou et al., 2006). ACGK is an unpublished assessment tool developed by the Centre for Sign Linguistics and Deaf Studies, Chinese University of Hong Kong. It aims to assess children's syntactic and morpho-syntactic knowledge of written Chinese that is based on Mandarin Chinese grammar. HKCOLAS-CG is a standardized tool for assessing children's grammatical knowledge of spoken Cantonese. All test items in ACGK are presented in written Chinese whereas HKCOLAS-CG requires children to listen and make responses in Cantonese. Since the Deaf children's speech perception abilities varied, the low scores that some achieved in HKCOLAS-CG may be due to the auditory mode of the assessment. Table 2 lists each participant's scores of ACGK and HKCOLAS-CG, which were obtained during the same time when the current study was conducted. Their speech perception scores were collected based on two Cantonese assessment tools, one for tone identification—*Cantonese Lexical Neighborhood Test (CLNT)* (Yuen et al., 2008) and the other one for disyllabic word recognition—*Cantonese Spoken Word Recognition Test (CanSWORT)* (Ng, 2014). Note also that Tang et al. (2014) reported a significant positive correlation not only between 20 SLCO Deaf children's developing grammatical knowledge of oral Cantonese and written Chinese (*r* = 0.790^**^, *p* = 0.000, 1-tailed); but also a positive interaction between HKSL and written Chinese (*r* = 0.591^**^, *p* = 0.003, 1-tailed) and between HKSL and oral Cantonese (*r* = 0.663^**^, *p* = 0.001, 1-tailed). The data analyzed in Tang et al. (2014) came from the same assessment tools mentioned here, including ACGK, HKCLOS-C as well as *Hong Kong Sign Language Elicitation Tool* (HKSL-ET). Meanwhile, all the DHs and DDs in the current study, except for 1 DH in Group 3 and 1 in Group 4, were subjects in Tang et al. (2014).

**Table 2:** Deaf children's performance on spoken languages.

**Participants**	**Research Code**	**ACGK (%)**	**HKCOLAS-CG (%)**	**CLNT (%)**	**CanSWORT (%)**
Group 1	DH-G1-1	95.19	70.83	100	95
	DH-G1-2	84.62	48.38	8	4.17
	DH-G1-3	81.73	41.67	0	0
	DH-G1-4	81.73	32.87	68	78.33
Group 2	DH-G2-1	69.23	43.98	4	0
	DH-G2-2	83.65	52.78	72	55.83
	DH-G2-3	89.42	42.82	96	41.67
	DH-G2-4	91.35	33.10	0	0
Group 3	DH-G3-1	93.27	70.14	92	93.33
	DH-G3-2	79.81	31.71	84	85.83
	DH-G3-3	88.46	67.59	96	49.17
	DH-G3-4	95.19	56.71	100	95
Group 4	DH-G4-1	62.50	39.35	92	60.83
	DH-G4-2	75.96	43.06	84	93.33
	DH-G4-3	97.12	78.70	92	76.67
DD	DD-1	96.15	75.23	96	91.67
	DD-2	92.31	66.67	92	100

In this experiment, three native Deaf signers (1 male and 2 female) participated as controls. All of them had two signing Deaf parents. They were 27-, 28-, and 33-year-old at the time of the experiment. Two of them graduated from the same school for the deaf that adopted the oral approach. The third one attended the same deaf school as the other two but transferred to a mainstream secondary school from Form 4 to Form 7.

### Materials and elicitation procedures

This study was part of a large-scale project approved by the Survey and Behavioral Research Ethics Committee (SBREC) at The Chinese University of Hong Kong. All the adult participants and parents of child participants signed a written, informed consent form. The child participants were individually tested in a quiet room at school while the adult participants were tested at the Centre for Sign Linguistics and Deaf Studies. Trained Deaf research assistants followed a strict protocol when administering the test battery, *Hong Kong Sign Language Elicitation Tool*, which is an unpublished assessment tool for profiling Deaf children's HKSL development in terms of production and judgments of grammaticality. The tool includes several subtests for different grammatical components, including classifier constructions, agreement verbs, negators, modals, *wh*-questions, *yes-no* questions, and non-manual adverbials.

**Figure 1:**
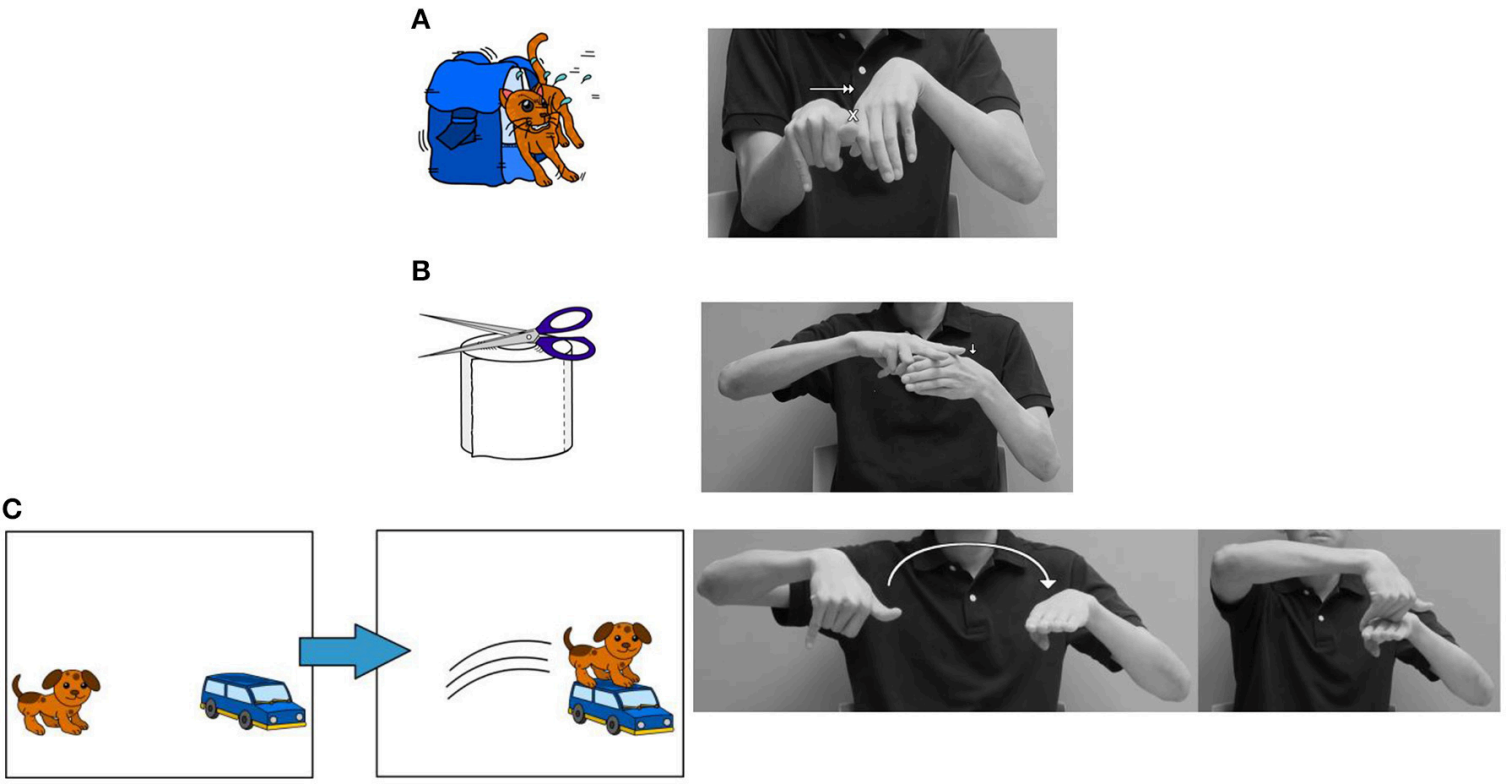
Pictures to elicit **(A)** transitive, **(B)** locative existential, and **(c)** motion directional classifier constructions.

The test on classifier constructions was a picture description task which took about 15 min to complete. In this task, all participants were asked to describe a set of 16 pictures in HKSL: six pictures for locative existential constructions, six for motion directional constructions and four for transitive classifier constructions. Figure 1 provides three sample pictures as stimuli for eliciting the different types of classifier constructions in the current study. The target HKSL sentences can be seen in examples (5a–c), while (7b–e) are the Cantonese counterparts. The experimenter showed the pictures one by one to the participants, who were allowed time to study the picture. Then, the experimenter removed the stimuli and the participants described the picture in HKSL. Additionally, a picture-naming task was conducted prior to the picture description task to control for vocabulary comprehension, as lexical variation is common among the HKSL signers, so a vocabulary check was necessary to ensure the participants' comprehension and production of the objects in the stimuli. The whole procedure was video-taped, and the participants' productions of the stimuli were transcribed using ELAN and coded accordingly.

As pointed out in the previous sections, a change of word order occurs with complex verb morphology in a classifier construction. In this study, all stimuli involved two arguments mapped onto a grammatical subject and object respectively. We selected different predicate roots, phonologically expressed by the dominant hand moving toward the non-dominant hand. In locative existential predicates, the locative root “be_located” requires a small downward movement toward a location argument. In motion directional predicates, three transfer roots—“jump onto,” “fall from,” and “fall onto”—were selected for the experiment. They require an “arc” path movement of the dominant hand from one R-locus to another R-locus that is occupied by the non-dominant hand. The transitive predicates also involve a transfer root translated as “push” and “press against.” It involves a path or orientation change of the dominant hand toward the non-dominant hand.

There are three types of classifiers in the predicates, coded based on Supalla (1982) categorization—semantic, SASS (i.e., size-and-shape specifiers), and bodypart classifiers. The semantic classifiers were used for co-reference with a dog, a cat, an elephant, a horse, and a toy car; SASSes for a rock, a backpack, a present, a toilet roll, and a pair of scissors; and bodypart classifier for a bionic hand. These classifiers were assigned to the dominant hand in the formation of a locative existential or motion directional predicate, where the non-dominant hand was either a semantic classifier or a SASS classifier. For the transitive predicates, only semantic and SASS classifiers were adopted. The classifiers on the dominant hand were all semantic, while the classifiers on the non-dominant hand were either SASS or semantic classifiers.

### Coding procedures

All production data were transcribed using ELAN (http://tla.mpi.nl/tools/tla-tools/elan/; Crasborn and Sloetjes, 2008) and coded with reference to a set of criteria based on reported analyses of HKSL. In this paper, two criteria were adopted in coding the children's performance. The first one was *verb root* of the main predicate, realized phonologically by the movement of the classifier on the dominant hand toward that on the non-dominant hand (henceforth MVR). The second one was *word order* (henceforth WO). We focused on these two criteria because we predict that properties of the verb root interact with word order changes in classifier constructions. Using the adults' performance as controls, the Deaf children's productions were categorized into adult-like performance and non-adult-like performance. The children's encoding of the predicates through gesture, lexical verbs, classifying verbs comprised of classifier handshapes was also coded. The data were scored by one Deaf researcher who is a native signer of HKSL, and one hearing researcher who is one of the co-authors of this paper. The rate of agreement between the two coders on the two criteria was 90%.

## Results

### Adult deaf signers

Data from three adult native Deaf signers formed the baseline of the current study. All of their responses showed adult-like classifier constructions in terms of target MVR and WO, except for one token of WO (see 8). Instead of one motion directional classifier construction, a male Deaf participant produced a serial verb construction made up of a locative existential classifier predicate and two motion directional classifier predicates. Such kind of serial verb constructions was seldom observed in the Deaf children's data. In all, data from the adult Deaf signers suggested the stimuli for the current study are sensitive to eliciting classifier constructions.

(8)
  **Table:** 

HKSL:	DH:	DOG_i_		SCISSORS_j_	be_located_on_b_+CL_SASSj_
	NDH:		be_located_a_+CL_SEMi_	————————->————————–	

	DH:	—————>————-		_b_fall_off_from_d_+CL_SASSj_	
	NDH:	_b_move_c_+CL_SEMi_		——————->—————	
		“A dog is located here; a pair of scissors is on (the back of) the dog; the dog moves and the pair of scissors falls off from (the back of) the dog”

As mentioned, while classifier constructions allow both OSV and SOV orders, the former order is much more common than the latter. This is confirmed by our adult signers' productions (Table 3). Over 94% of the tokens produced were of an OSV order (Table 3). Only 1 token of SOV order with a locative existential predicate was found.

**Table 3:** Performance on WO by adult Deaf signers.

**Predicates**	**O > S > V (%)**	**S > O > V (%)**	**Serial verbs (%)**	**Total no. of responses (*N* = 3)**
Transitive	12 (1.00)	–	–	12
Loc-exist	17 (.94)	1(0.06)	–	18
Mot-dir	17 (.94)	–	1 (0.06)	18

### Deaf children

Using the results from the adult signers as baseline, we coded the responses as non-adult-like performance when no classifier construction was produced by the DHs. One DH from Group 1 actually produced three tokens of transitive predicates in an SVO order with no classifier constructions (see 9); however, these sentences were coded as grammatical. According to the Deaf rater, the child used role shift together with an inflected agreement verb PUSH in the main predicate. Therefore, we removed these 3 tokens from our analysis. Table 4 summarizes the distribution of the total number of responses (Group x Types of classifier constructions x Number of tokens). Subsequent analyses presented below adopt these numbers as denominators in the calculation.

(9)
  **Table:** 

		______rs			
HKSL:	CAT_a_	_a_PUSH_b_	IX_b−j_		ELEPHANT_b−j_
	“A cat pushes an elephant.”				(DH-G1-3)

**Table 4:** Number of responses for the current analyses*.

**Participants**	**Transitive**	**Loc-exist**	**Mot-dir**	^*^/Total number of responses (%)
Group 1 (*N* = 4)	13	24	24	61/64 (0.95)
Group 2 (*N* = 4)	16	24	24	64/64 (1.00)
Group 3 (*N* = 4)	16	24	24	64/64 (1.00)
Group 4 (*N* = 3)	12	18	18	48/48 (1.00)
DD (*N* = 2)	8	12	12	32/32 (1.00)

Generally, while all tokens of WO from the DDs were adult-like (i.e., 100%), only 66% of their MVR tokens were adult-like (see Table 5). As for the DHs, the numbers of adult-like tokens of MVR and WO of Group 1 were similar to those DDs (i.e., MVR = 74%; WO = 92%), suggesting the possibility of achieving near-native competence in classifier constructions. On the other hand, the number of adult-like responses for MVR and WO dropped from Groups 2 to 4. Group 2's adult-like responses were 56% for MVR and 58% for WO; Group 3 were 39% for MVR and 22% for WO; and Group 4 were 46% for MVR and 21% for WO. These results suggest that duration of exposure to HKSL has an effect on their acquisition. Also, Groups 3 and 4's performance on WO implies that word order changes in classifier constructions posed initial difficulty. In the following two sections, we will describe the participants' performance on MVR and WO.

**Table 5:** Production of adult-like MVR/WO by DHs and DDs.

**Participants**	**Predicates**	**Adult-like MVR**	**Group Total (%)**	**Adult-like WO**	**Group Total (%)**	**Total no. of responses**
Group 1 (*N* = 4)	Transitive	10	45 (0.74)	12	56 (0.92)	61
	Loc-exist	11		22		
	Mot-dir	24		22		
Group 2 (*N* = 4)	Transitive	9	36 (0.56)	9	37 (0.58)	64
	Loc-exist	3		12		
	Mot-dir	24		16		
Group 3 (*N* = 4)	Transitive	0	25 (0.39)	4	14 (0.22)	64
	Loc-exist	1		6		
	Mot-dir	24		4		
Group 4 (*N* = 3)	Transitive	4	22 (0.46)	1	10 (0.21)	48
	Loc-exist	0		1		
	Mot-dir	18		8		
DD (*N* = 2)	Transitive	5	21 (0.66)	8	32 (1.00)	32
	Loc-exist	4		12		
	Mot-dir	12		12		

### Deaf children's performance on MVR

As mentioned, the verb root of a classifier predicate is morphologically different from the other types of verbs in sign languages. Table 6 shows the distribution of adult-like MVR responses over the three types of classifier constructions. While almost all DHs reached the ceiling of performance on MVR in motion directional predicates, their production of adult-like MVR in transitive and locative existential predicates dropped dramatically. The MVR of locative existential predicates turned out to be the most difficult for all children, including the DDs^8^. As shown in Table 6, a great majority of them, especially those in Groups 3 and 4, either failed to produce a classifier predicate and used other lexical verbs (e.g., HAVE) or failed to realize the verb “be_located” using a small downward movement. In the latter case, they adopted a long downward movement which bears other predicate meanings (also see data description below). Previous studies argued that due to iconicity, not only DDs but also DHs can spatially encode the locative relation between a Figure and a Ground as early as age 2;0 (Lindert, 2001). The current findings suggest locating them at specific R-loci in space through a specific movement feature turned out to be quite difficult. We argue that it is due to their not selecting the locus features from List 1 initially, and at the same time not realizing that the properties of movement are morphemic.

**Table 6:** Production of adult-like MVR by DHs and DDs.

**Predicate types**	**Transitive**		**Loc-exist**		**Mot-dir**		**Adult-like responses/total responses per group (%)**
	**Adult-like responses (%)**	**Total responses**	**Adult-like responses (%)**	**Total responses**	**Adult-like responses (%)**	**Total responses**	
Group 1	10 (0.77)	13	11 (0.46)	24	24 (1.00)	24	45/61 (0.74)
Group 2	9 (0.56)	16	3 (0.13)	24	24 (1.00)	24	36/64 (0.56)
Group 3	0 (0.00)	16	1 (0.04)	24	24 (1.00)	24	25/64 (0.39)
Group 4	4 (0.33)	12	0 (0.00)	18	16 (.89)	18	20/48 (0.42)
DD-1	1 (0.25)	4	2 (0.33)	6	6 (1.00)	6	9/16 (0.56)
DD-2	4 (1.00)	4	2 (0.33)	6	6 (1.00)	6	12/16 (0.75)

**Figure 2:**
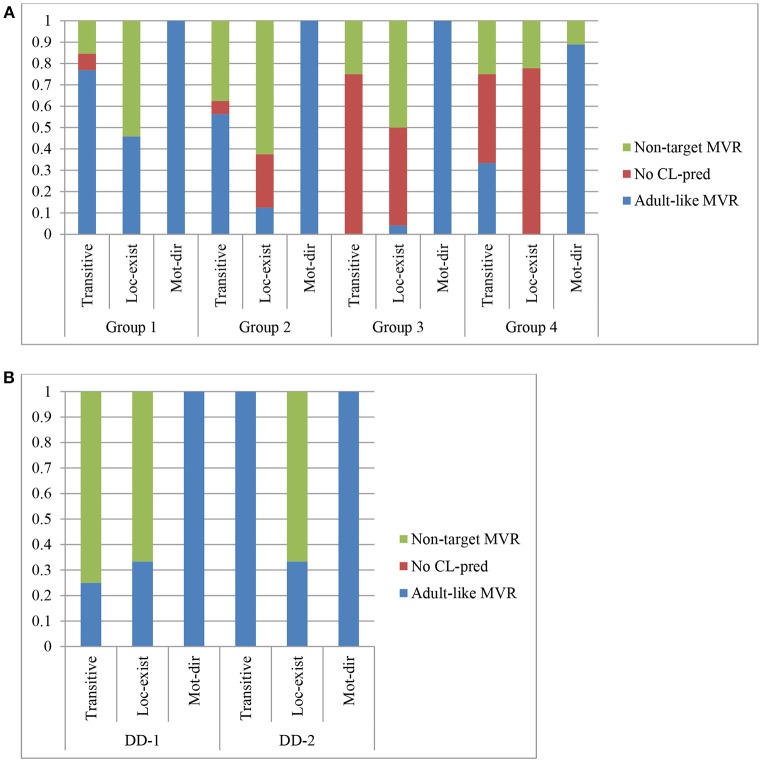
Production of adult and non-adult-like MVR by DHs **(A)** and DDs **(B)**.

To further analyze group performance on the verb root, we categorized the DHs' errors into two types (see Figures 2A,B and Table 7). The first type of errors shows the DHs' lack of production of classifier predicates (i.e., “No CL-pred”). As shown, such a lack was observed only in transitive and locative existential predicates but not motion directional predicates, especially among children from Groups 3 and 4. These children selected an equivalent lexical verb instead if they could identify one, such as PUSH in (10a) and HAVE in (10b). Note that the agreement verb PUSH in the context of an ordinary transitive predicate requires an SOV/OSV order or in SVO order with role shift, as in (9). However, none of such word orders or role shift with SVO order was observed in the DH's productions.

(10a)
  **Table:** 

^*^HKSL:	CAT	PUSH	ELEPHANT	
	“A cat pushes an elephant.”			(DH-G3-4)

(10b)
  **Table:** 

^*^HKSL:	TOILET^∧^ROLL	IX_up_	HAVE	SCISSORS	
	“A pair of scissors is on the toilet roll.”				(DH-G4-2)

**Table 7:** Production of non-adult-like MVR by DHs and DDs.

**Predicate types**	**Transitive**		**Loc-exist**		**Mot-dir**	
**Error types**	**No CL-pred (%)**	**Non-target MVR (%)**	**No CL-pred (%)**	**Non-target MVR (%)**	**No CL-pred (%)**	**Non-target MVR (%)**
Group 1	1 (0.08)	2 (0.15)	0 (0.00)	13 (0.54)	0 (0.00)	0 (0.00)
Group 2	1 (0.06)	6 (0.38)	6 (0.25)	15 (0.63)	0 (0.00)	0 (0.00)
Group 3	12 (0.75)	4 (0.25)	11 (0.46)	12 (0.50)	0 (0.00)	0 (0.00)
Group 4	5 (0.42)	3 (0.25)	14 (0.78)	4 (0.22)	0 (0.00)	2 (0.11)
DD-1	0 (0.00)	3 (0.75)	0 (0.00)	4 (0.67)	0 (0.00)	0 (0.00)
DD-2	0 (0.00)	0 (0.00)	0 (0.00)	4 (0.67)	0 (0.00)	0 (0.00)

In total, there were 50 tokens of MVR errors under the category of “No CL-pred” among which a majority of them (43 out of 50 tokens, 86%) showed a SVO order and involved either a lexical verb or gesture (see section SVO Order With a Variety of Verb Roots, Table **10**). It is obvious that these children resorted to selecting a lexical verb root initially, and the lower the grades the higher the percentages of such erroneous productions. Therefore, so far as the transitive predicates and locative existential predicates are concerned, Deaf children from the lower grades tended to select, from List 1, a lexical root but not features pertaining to a classifier construction in the Numeration.

**Figure 3:**
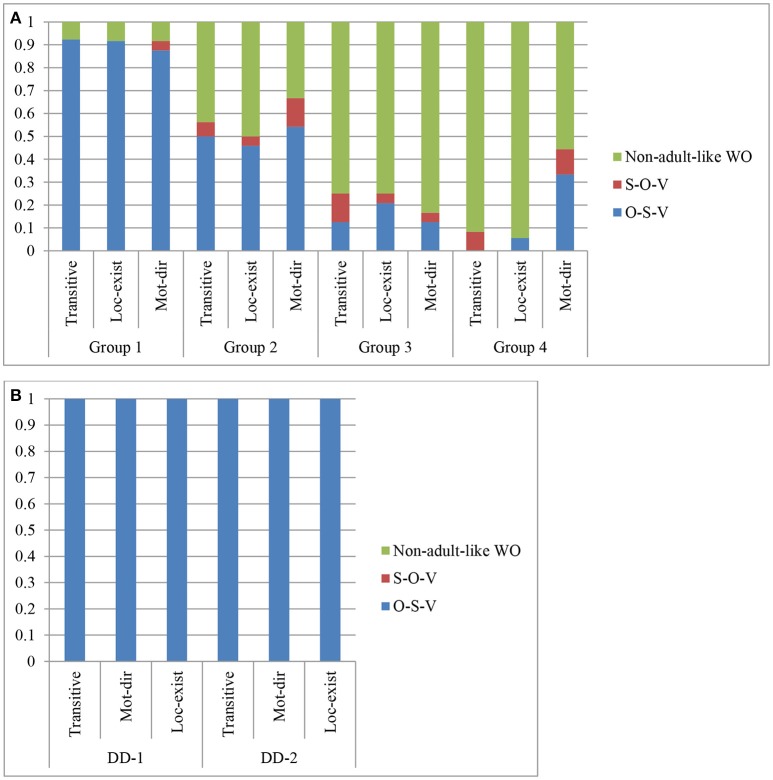
Production of adult and non-adult-like WO by DHs **(A)** and DDs **(B)**.

The second type of errors is related to how children encode events or states realized by movement (i.e., verb root) with classifier morphemes. In our analysis, we assumed such errors were morpho-phonological (i.e., “Non-target MVR” in Figure 3 and Table 7), and were generally found in the locative existential predicates. In fact, all the errors produced by the DDs belonged into this category. For the native Deaf adults we consulted, these non-target MVRs encode a different predicate meaning. As said above, most DHs and DDs produced a long downward movement for locative existential predicates instead of the target which is a small downward movement. Such a long downward movement signals three different meanings: “fall down from (a high position),” “put something at (a location),” and “jump onto” a location. Among the 44 tokens of such errors extracted from the locative existential predicates, about 28 of them produced by the DHs had a meaning of “put something at (a location),” and 7 such tokens were accompanied by mouthing the Cantonese verbs *fong3* or *baai2* “put.” This finding suggests that, instead of selecting an abstract HKSL verb root “be_located,” these children preferred to select a lexical, locative verb like *fong3* in Cantonese (e.g., *baa2 gaau3zin2* ***fong3 hai6*** *gyun2 ci3zi2 soeng6min6* “The scissors are (placed) on top of the toilet roll”).

In sum, the findings of MVR reveal that Deaf children experienced initial difficulty in selecting an abstract verb root for the classifier predicates in HKSL. Before converging on the adults' grammar, we observed a lack of use of classifier predicates, especially in locative existential predicates, and insertion of a lexical verb root was the usual strategy, if they could identify one. Also, adopting an appropriate movement shape to encode the existential verb root led to morphophological errors in their production. In the next section, we proceed to analyze how Deaf children's knowledge of verb root interacts with their acquisition of word order.

### Deaf children's performance on word order

As said, while OSV and SOV are the two acceptable word orders of classifier constructions in HKSL, elicited data from three native adult signers showed that OSV order was more prevalent, except for 2 tokens (see Table 3). As for the Deaf children, Table 8 shows that the two DDs produced adult-like word order consistently. Additionally, 117 out of 237 responses of the DHs were adult-like; and those DHs with longer exposure to HKSL produced more tokens of adult-like word order for all three types of classifier constructions. Group 1 reached almost the ceiling of performance (i.e., 92%), Group 2 between 50 and 67%, but Groups 3 and 4 had much fewer adult-like WOs for all three types of classifier constructions.

**Table 8:** Production of adult-like WO by DHs and DDs.

**Predicate types**	**Transitive**		**Loc-exist**		**Mot-dir**		**Adult-like responses /total responses per group (%)**
	**Adult-like responses (%)**	**Total responses**	**Adult-like responses (%)**	**Total responses**	**Adult-like responses (%)**	**Total responses**	
Group 1	12 (0.92)	13	22 (0.92)	24	22 (0.92)	24	56/61 (0.92)
Group 2	9 (0.56)	16	12 (0.50)	24	16 (0.67)	24	37/64 (0.58)
Group 3	4 (0.25)	16	6 (0.25)	24	4 (0.17)	24	14/64 (0.22)
Group 4	1 (0.08)	12	1 (0.06)	18	8 (0.44)	18	10/48 (0.21)
DD-1	4 (1.00)	4	6 (1.00)	6	6 (1.00)	6	16/16 (1.00)
DD-2	4 (1.00)	4	6 (1.00)	6	6 (1.00)	6	16/16 (1.00)

#### OSV order as the preferred word order

Figures 3A,B as well as Table 9 show the different word order produced by the DHs. Similar to the native adults, all of the 32 WO responses produced by the DDs reflected the adult-like OSV order (see Figure 3B). Figure 3A shows that there was a big tendency for the OSV order in the DH's adult-like productions. This preference was observed even among the DHs of Groups 3 and 4. In fact, there were very few tokens of SOV order in the data, suggesting that it was a much less preferred order among the DDs and DHs. Also, the production of an OSV order for classifier constructions by Deaf children, as we argue, is taken to be evidence that they are selecting the locus features for the classifiers in the Numeration, for them to assign the classifier for the grammatical object to an R-locus in space through an initial locative existential predicate.

**Table 9:** Production of OSV and SOV orders of DHs and DDs based on adult-like responses.

**Predicate types**	**Transitive**		**Loc-exist**		**Mot-dir**	
	**O-S-V (%)**	**S-O-V (%)**	**O-S-V (%)**	**S-O-V (%)**	**O-S-V (%)**	**S-O-V (%)**
Group 1	12 (0.92)	0 (0.00)	22 (0.92)	0 (0.00)	21 (0.88)	1 (0.05)
Group 2	8 (0.50)	1 (0.06)	11 (0.46)	1 (0.04)	13 (0.54)	3 (0.13)
Group 3	2 (0.13)	2 (0.13)	5 (0.21)	1 (0.04)	3 (0.13)	1 (0.04)
Group 4	0 (0.00)	1 (0.08)	1 (0.06)	0 (0.00)	6 (0.33)	2 (0.11)
DD-1	6 (1.00)	0 (0.00)	6 (1.00)	0 (0.00)	6 (1.00)	0 (0.00)
DD-2	6 (1.00)	0 (0.00)	6 (1.00)	0 (0.00)	6 (1.00)	0 (0.00)

#### Non-adult-like responses

##### SVO order with a variety of verb roots

**Figure 4:**
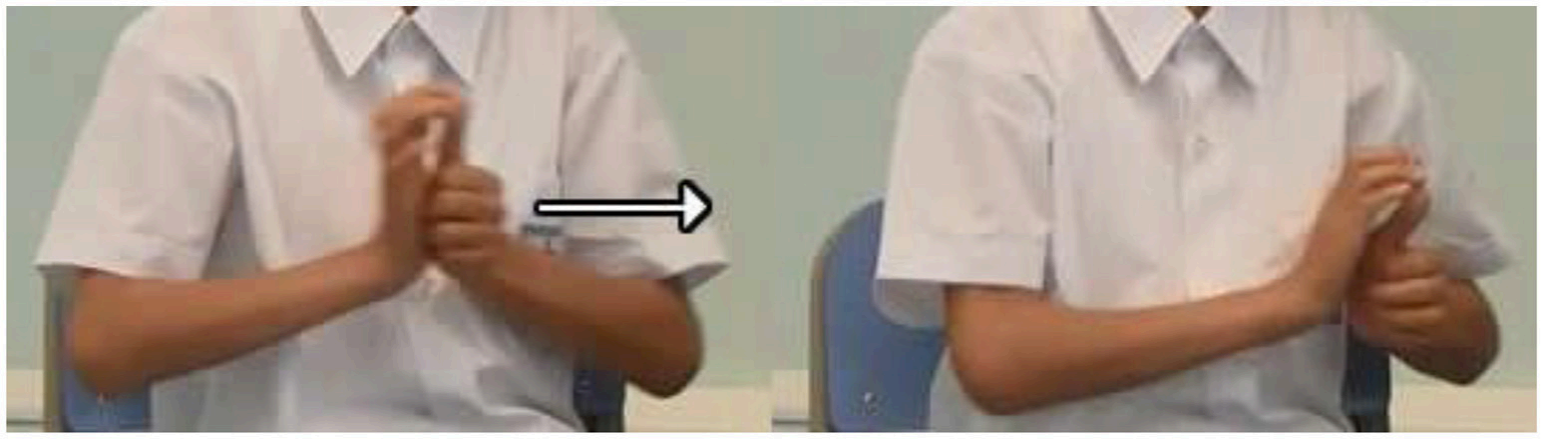
An example of V_complex_ meaning “the cat pushes the backpack”.

Among the 120 non-adult-like productions out of the 237 responses produced by the DHs, 91 (i.e., 76%) reflected a clear SVO order which is not acceptable for classifier constructions. In fact, it is difficult to determine if the knowledge of SVO order stems from Cantonese or HKSL, as both languages allow SVO as the basic word order, as discussed previously (see section Crosslinguistic Comparison and Acquisition Predictions). Yet, the way these children inserted the verb root into this basic SVO structure deserves our attention. We found 5 types of “verb roots” from their production (see Table 10). 68% of such errors belonged to either uninflected lexical verbs (i.e., V_lexical_) or a form of two-handed signs which did not resemble a lexical sign. They were usually configured by two inappropriate classifier-like handshapes (i.e., V_complex_, see Figure 4) and without spatial information. We took such productions to be morphologically complex signs but non-target both in terms of handshape configuration and spatial information. Other types of verb roots were just 4 tokens of gesture [see (11) produced by DH-G3-1], 2 tokens of verb series V_complex_+ V_lexical_, and 2 tokens of a one-handed motion directional predicate. Following Language Synthesis, the S >V_lexical_ > O structure represents an output based on selecting the morphosyntactic features pertaining to a lexical verb root without classifier or locus features leading to PUSH or PUT in Vocabulary Insertion. This phenomenon occurred more frequently with the DHs in Group 3 and 4 but gradually dropped upon longer duration of exposure to HKSL.

(11)
  **Table:** 

^*^HKSL:	SMALL^∧^CAT	gesture [ = push by shoulders]	HEAVY	ELEPHANT
	“A small cat pushes (itself against) a heavy elephant.”			(DH-G3-1)

**Table 10:** Non-adult-like occurrences of word order and verb root.

**Erroneous patterns**	**SVO with a variety of verb roots: 91/120 (76%)**					
	**S** > **V**_lexical_ > **O**	**S** > **V**_complex_ > **O**	**S** > **ges** > **O**	**S** > **V**_complex_+ **V**_lexical_ > **O**	**S** > **1-handed Mot-dir CL** > **O**	**Total**
Group 1	1	2	0	0	0	3
Group 2	6	18	0	0	0	24
Group 3	15	13	2	1	2	34
Group 4	17	9	2	1	2	30
Total	39	42	4	2	4	91

**Figure 5:**
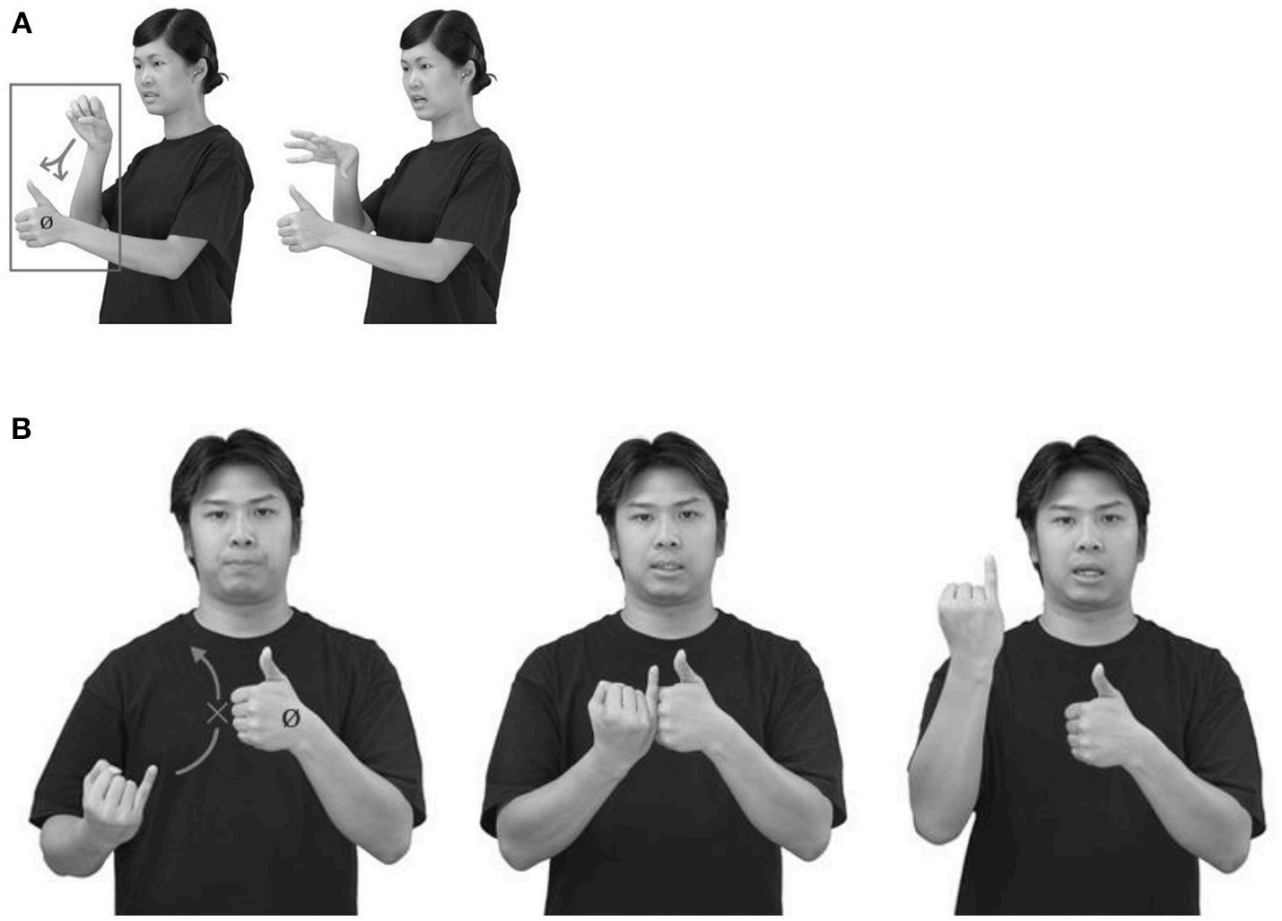
**(A)** SCOLD, **(B)** REBEL.

While V_lexical_ predominated the data of Groups 3 and 4, V_complex_ showed an almost reverse pattern of distribution, in the sense of an increasing tendency of production when the DHs moved up to Grades 2 and 1. In Group 1, the DHs knew SVO with a V_complex_ was ungrammatical in HKSL, as evidenced by the production of just two tokens of S > V_complex_ > O. In other words, the production of a V_complex_ sign during the initial acquisition process did not necessarily trigger reordering of SVO, contrary to our prediction. One reason is that when knowledge of SVO order based on a lexical verb root is doubly enhanced by Cantonese and HKSL, these children might wrongly assume that verbs are paradigmatically lexical in nature. Another reason may stem from ambiguous input. We suspect that the DHs from Groups 2, and especially 3 and 4, might initially produce these V_complex_ signs as “lexical signs,” similar to those two-handed lexical verb signs like SCOLD or REBEL in HKSL (Figure 5), which do not bear any locus or classifier features although they have a classifier predicate origin. Therefore, the erroneous constructions suggest that projections for object agreement which triggers word order changes were not in place yet, due to the absence of locus features despite the presence of classifier features. Consequentially, the word order remained as SVO as no formal agreement relation was established between the verb and the R-loci (see Discussion below).

Table 11 offers a further analysis of the distribution of the two major non-adult-like verb roots, V_lexical_ and V_complex_, in SVO order. The data are organized based on the DHs' performance on the types of classifier predicates by groups.

**Table 11:** Distribution of V_lexical_ and V_complex_ in a non-adult-like SVO order^*^.

**V_lexical_ and V_complex_ in SVO**	**Transitive**			**Loc-exist**			**Mot-dir**		
	**V**_lexical_ **(%)**	**V**_complex_ **(%)**	Total^*^	**V**_lexical_ **(%)**	**V**_complex_ **(%)**	Total^*^	**V**_lexical_ **(%)**	**V**_complex_ **(%)**	Total^*^
Group 1	1 (1.00)	0 (0.00)	1	0 (0.00)	0 (0.00)	2	0 (0.00)	2 (1.00)	2
Group 2	1 (0.14)	6 (0.86)	7	5 (0.42)	5 (0.42)	12	0 (0.00)	7 (0.88)	8
Group 3	8 (0.67)	0 (0.00)	12	7 (0.39)	3 (0.17)	18	0 (0.00)	10 (0.50)	20
Group 4	3 (0.27)	2 (0.18)	11	14 (0.82)	2 (0.12)	17	0 (0.00)	5 (0.50)	10

(^*^ Total numbers of erroneous responses as denominators).

As for V_lexical_, uninflected PUSH is selected consistently in transitive predicates. For locative existential predicates, we found a variety of lexical verbs such as uninflected PUT and main verb HAVE (see 12, 13a). These verbs were usually accompanied by a pointing sign IX_up_ that served more like a Cantonese localizer *soeng6min6* “up.” The use of HAVE, as in (13a), has a meaning similar to the existential verb *yau5* “have” in Cantonese [see (13b) for the Cantonese counterpart], suggesting that the structure with PUT or HAVE is based on the Cantonese SVO order (see next section).

Note that in Table 11, we found no records of DHs across all groups inserting a V_lexical_ into a motion directional predicate in HKSL. The lack of equivalence in the morphosyntactic structure of verb roots between HKSL and Cantonese may be at play here. In HKSL, the verb root is expressed morphophonologically by a single path movement, which also iconically maps the path between the source and the goal arguments; however, Cantonese's motion directional predicates require serial verb constructions, such as *tiu3soeng6heoi3* (lit. “jump ascend go”), *dit3lok6lei4* (lit. “fall descend come”). It is interesting to observe that the DHs seemed to be sensitive to such differences early on, as evidenced by a high instance of correct MVR tokens (see Figure 2A).

(12)
  **Table:** 

^*^HKSL:	IX_i_	SCISSORS_i_	PUT	IX_j_	TOILET^∧^ROLL_j_	IX_up_
	“A pair of scissors is on a toilet roll.”					(DH-G4-3)

(13a)
  **Table:** 

^*^HKSL:	DOG	IX_up_	HAVE	BIONIC^∧^HAND	
	“A bionic hand is on a dog.”				(DH-G3-4)

(13b)
  **Table:** 

Cantonese:	zek3	gau2	soeng6min6	**yau5**	zek3	gei1hai6sau2
	CL	dog	(on the) top of	have	CL	bionic hand
	“A bionic hand is on a dog.”

Turning to V_complex_, as said, they are composed of two classifier-like handshapes with a movement to represent the verb root, as in (14). In an SVO context, it occurred mostly in locative existential and motion directional predicates, except for the DHs of Group 2 and Group 4 who also produced 6 and 2 such tokens in transitive predicates respectively. This V_complex_, which shows some properties of a classifier predicate, may reflect the DHs' initial knowledge of representing the argument relation of the noun referents in an event or a state only. However, it is not associated with abstract morphosyntactic features for referentiality, spatial or subject/object agreement; otherwise, OSV order should occur in their performance, recalling in Table 9 that OSV only began to occur systematically from Group 2 onwards.

(14)
  **Table:** 

^*^HKSL:	DH:	DOG	V_complex_: jump_onto	STONE	
	NDH:				
		“A dog jumps onto a rock.”			(DH-G2-2)

To conclude, before attaining native or near-native competence as what the DHs of Group 1 managed to achieve, the DHs of Groups 2, 3 and 4 would initially assume an SVO order with a V_complex_ or a V_lexical_ for the three types of classifier predicates. These data suggest evidence of language interaction effects in the domains of word order and verb root. The SVO stage may stem from crosslinguistic influence from Cantonese and/or the DHs' internal developing HKSL grammar of SVO order with a lexical verb root. However, the observation that more DHs in the senior groups embedded a V_complex_ in an SVO or OSV order suggests their increasing morphosyntactic knowledge of this complex predicate, thereby triggering agreement and subsequent syntactic operations like topicalization of the object argument in a construction involving a locative existential predicate.

##### Other mixed structures

For the remaining 29 out of 120 tokens of non-adult-like responses that could not be grouped into a straightforward SVO category, we call them “mixed structures” because in some cases we observed mixing of grammatical properties of Cantonese and HKSL in the derivation, in other words, there is the possibility of mixed Numeration (see Table 12). We discarded one token due to our failure of comprehending the string of signs produced by a DH from Group 3.

**Table 12:** Occurrences of other mixed structures.

**Error patterns**	**Other Mixed Structures: 29/120 (24%)**			
	**Cantonese structure**	**Pointing signs as localizers**	**Discard**	**Total**
Group 1	0	2	0	2
Group 2	1	2	0	3
Group 3	9	7	1	17
Group 4	7	0	0	7
Total	17	11	1	29

##### Cantonese-based structure

Seventeen tokens were grouped under this category. Fourteen tokens came from a structure in which the first part of the sentence is contributed by the Cantonese grammar but the final verbal predicate is from HKSL. As shown in (15a), the subject DOG, a location argument, is marked by a pointing sign IX_up_ equivalent to a localizer in Cantonese. It is followed by the main verb HAVE which is also similar to Cantonese *jau3* with an existential meaning, and the object SCISSORS, hence reflecting an SVO order. The second verb is a V_complex_, comprised of two classifiers to encode a motion directional predicate (i.e., a pair of scissors fall down from the back of a dog). In fact, this string SV_HAVE_ OV_complex_ suggests a derivation based on Cantonese grammar (see 15b); yet, a V_complex_ is inserted into the second verb slot at Vocabulary Insertion. Note that 11 out of these 14 tokens of V_complex_ displayed an adult-like movement shape to denote a motion directional or a transitive predicate, suggesting that this clause final V_complex_ is more like a classifier predicate.

(15a)
  **Table:** 

^*^HKSL:	DH:	DOG	IX_up_	HAVE	SCISSORS	**V_complex_: _a_fall_down_b_**	
	NDH:		———————>—————————				
		“There is a pair of scissors on a dog, (the scissors) falls down(from the back of the dog).”					(DH-G4-1)

(15b)
  **Table:** 

Cantonese:	gau2	soeng6min6	jau3	gaau3zin2	dit3 lok6lei4
	dog	on top of	have	scissors	fall down
	“A pair of scissors fall down from the back of the dog.”

##### Pointing signs as localizers

The second group of data displaying a mixed Numeration came from 3 tokens of utterances produced by the DHs from Groups 3 and 4. The utterances were derived based on the word order of existential predicates in Cantonese but the verb root “be_located” in HKSL or *hai2* in Cantonese was missing. In place of it, we observed a pointing sign (see 16). *Hai2* in Cantonese is seldom found even in Cantonese-based signing. Therefore, resorting to pointing signs enabled them to encode the locative relation of the two arguments.

(16)
  **Table:** 

^*^HKSL:	DH:	SCISSOR		LITTLE^∧^DOG	IX_BACK_	IX_up_
	NDH:				—->—-	

Cantonese base:		gaau3zin2	(hai2)	zek3 siu2gau2	bui3	soeng5
		scissors	(be located)	CL small.dog	back	up
		“There is a pair of scissors on a dog's back.”				(DH-G3-1)

Six tokens of locative existential and two tokens of motion directional classifier constructions were nearly adult-like, except that a pointing sign (e.g., IX_up_ or IX_back_) was inserted to serve more like a localizer for the location argument, which is redundant in HKSL (see 17).

(17)
  **Table:** 

^*^HKSL:	DH:	STONE_a_	BIONIC^∧^HAND	_a_IX_up_	V_complex_: _a_be_located_b_
	NDH:		—————>————–		
		“A bionic hand is on a rock.”			(DH-G2-1)

In summary, the data reveal that Deaf children acquiring classifier constructions in HKSL could converge on the adults' grammar after 6 to 7 years of exposure. Over time, they could assign a classifier to an R-locus in space using a locative existential predicate, which serves as the grammatical object for the ensuing transitive, locative existential and motion directional predicate for which the classifier on the dominant hand serves as subject. Before attaining this stage of knowledge, we observe evidence of crosslinguistic interaction between Cantonese and HKSL which we will discuss below.

## Discussion

One aim of the current study was to investigate if HKSL-Cantonese DHs, aided or implanted, whose onset of HKSL exposure was not at birth but at age 4 or even as late as age 6 or 7, managed to acquire the complex morphosyntactic properties of classifier constructions. Unlike the Kodas or DDs, their parents are not signers, and the SLCO environment is the only source of HKSL input. The findings show that, despite relatively late exposure to HKSL, these children are able to produce classifier constructions based on an OSV order with R-loci for the classifiers, for subject/object agreement as well as spatial agreement. In other words, the SLCO environment, designed to provide dual language input, especially HKSL from Deaf teachers and a critical mass of Deaf students on a daily basis, to some extent offsets the lack of HKSL input in the home environment. In addition to consistent HKSL input and duration of exposure, one other possibility is the Cantonese (and/or written Chinese) input in the SLCO environment, which bolsters bimodal bilingual acquisition and indirectly raises their metalinguistic awareness about differences in word order and verb morphology between Cantonese and HKSL, as well as other properties like the use of space to encode formal grammatical properties like referentiality and agreement (Tang et al., 2015). What we observed among these DHs is the initial adherence to the canonical SVO order and choice of lexical verb root, a property shared by both HKSL and Cantonese. Such a similarity in the morphological property of verbs actually invites crosslinguistic interaction between the two languages, leading to interesting developmental consequences, an issue which we attempt to account for using Language Synthesis.

When predicting effects of crosslinguistic interaction in the current study, we argue that word order and the morphosyntactic properties of the verb are the two domains in which such evidence may be found. The findings reveale that the DHs underwent a protracted SVO stage. During this period, they inserted either a lexical verb (i.e., V_lexical_) or a two-handed verbal sign (i.e., V_complex_) into this SVO structure. Such patterns were quite prominent among the DHs of Groups 3 and 4, especially in transitive predicates and locative existential predicates. Examining their non-adult-like tokens, we observed a frequent use of uninflected PUSH for the transitive predicates and HAVE for the locative existential predicates. These verbs have a lexical root which can easily find a translation equivalent in Cantonese such as *toei1* “push” and *jau5* “have.” Take the locative existential predicates as an example, 20 out of 25 non-adult-like tokens adopted HAVE in the predicate. In fact, HAVE in HKSL can be a verb of possession (e.g., KENNY DOG HAVE “Kenny has a dog”), an auxiliary verb encoding perfective aspect of an event (e.g., LAST EVENING IX-1 RUN ONE^∧^HOUR HAVE “Last evening I ran for 1 h”), and as a verb of existence (e.g., *HOUSEa IXa DOG HAVE*). Clearly, the syntactic position of HAVE is clause-final in the adult's grammar. However, in all these non-adult-like tokens, HAVE occurs in an SVO structure, which is similar to the existential verb *jau5* in Cantonese, as shown in (7d) above. Therefore, we argue that these children selected the morphosyntactic features of Cantonese initially from List 1, and at Vocabulary Insertion HAVE was selected from HKSL instead. Another piece of evidence for Cantonese influence is the insertion of a post-nominal pointing sign IX_up_ [e.g., in (17) above], which is reminiscent of a Cantonese localizer *soeng6min6* “(on the) top of” to encode the locative relation between two entities (e.g., “a dog on a rock”); however, it is redundant with a locative existential predicate in HKSL. Following Distributed Morphology, we assume it is the assembly of the morphosyntactic features pertaining to Cantonese *jau5* and localizer in the Numeration that determines the syntactic word order, although HAVE from HKSL can be chosen at Vocabulary Insertion. The protracted SVO stage could be a result of DHs not selecting classifier features and locus features in the Numeration initially, as they assumed HKSL verbs are similar to Cantonese which are lexical in nature. As a consequence, the syntactic derivation yields a canonical SVO order and Vocabulary Insertion selects lexical verbs that overlap in Cantonese and HKSL, such as HAVE or uninflected PUSH without subject/object agreement or spatial agreement.

The case of V_complex_ is a little complicated. As said, during this protracted SVO stage of development, we also found an increasing number of tokens of two-handed V_complex_ alongside the V_lexical_. Arguably, features for classifiers are selected and spelt out as a two-handed V_complex_, leading to a change of the morphological structure of the verb. However, other features, especially the locus feature for spelling out the R-loci for spatial agreement, are not selected initially. In other words, without the locus feature, no object agreement node is projected above little *v*P. At PF, it is the lack of R-loci for spatial agreement rather than classifier agreement that marks the V_complex_ distinct from those observed in the classifier predicates produced by the adult signers. Subsequent acquisition of classifier predicates, in particular the selection of locus feature for spatial agreement will lead to a further reanalysis of the morphological status of V_complex._ Such reanalysis triggers decomposition of the two-handed signs and the copying of classifier and locus features to different agreement nodes for structural agreement with the noun arguments at the specifier positions. Furthermore, the development of pragmatic knowledge involved in the signing discourse by these children also led to the object being introduced independently by a locative classifier predicate or probably through a movement operation to the left periphery. We leave this part of the analysis for future research.

Although our research is not particularly geared toward analyzing code blending, some of our data like those discussed above resemble what (Branchini and Donati, 2016) would refer to as Type 1 (i.e., Cantonese or HKSL-based) or Type 3 (i.e., mixed) structures. As for Type 3, as said, we found some mixed use of HKSL and Cantonese grammars. For instance, an additional verb of existence HAVE reflecting the Cantonese grammar is adopted to introduce a noun referent (usually the Figure), which is followed by V_complex_ at the clause-final position (see descriptions above under “Cantonese-based Structure”). In fact, 14 such erroneous mixed structures were produced by the DHs of Group 3 (i.e., 3 tokens for locative predicates and 4 tokens for motion directional predicates) and Group 4 (i.e., 1 token for locative predicates, 3 tokens for motion directional predicates, and 3 tokens for transitive predicates). In Cantonese, *jau5* “have”+NP introduces a theme argument whereas in HKSL it is introduced by a locative classifier predicate or some localization strategies. Therefore, what we believe to be evidence of a mixed structure came from the erroneous productions like (15a). Although the DHs adopted a Cantonese SVO structure, they attached a clause-final classifier predicate after the object. In other words, the lack of a direct translation equivalent for a lexical locative existential *hai2* “be located” to be signed in such a way that it becomes head final simply goes against the Cantonese grammar whose verbs are consistently head initial. As for the motion directional predicates, if the DHs followed the Cantonese grammar entirely, they had to produce three independent signs (i.e., VVV) due to serial verb constructions which uniquely occur in Cantonese but not in HKSL so far as motion directional predicates are concerned. That the DHs were in a test condition for HKSL production encouraged them to switch to the HKSL structure and choose a V_complex_ in some spatial configuration with a path movement with two endpoints to encode the source and the goal of the predicate. This finding also gives us some clues as to why they performed better on motion directional predicates than other predicates in the current study.

To sum up, this study reveals that Deaf children undergoing bimodal bilingual acquisition showed co-activation of the two grammars in the Numeration, during which they assumed knowledge of word orders available from the two languages, and the so called “mixing” occurred primarily in the verbal domain in their outputs. Among all the features they need to acquire for classifier constructions, the results show that locus features were acquired last in the process.

## Conclusion

Although earlier studies showed that classifier predicates may emerge more or less the same time as agreement verbs, full mastery was consistently reported to be late, owing to their morphosyntactic complexity. The current study revealed that consistent HKSL input over time could lead to convergence on the adult's grammar, despite a lack of early exposure to the language since birth. Where the home environment does not facilitate sign language acquisition, the school environment with consistent HKSL input from Deaf adults and Deaf peers becomes crucial for supporting the DHs' HKSL development. This echoes the findings from some previous studies that consistent sign language exposure in schools facilitates Deaf children's sign language development (Henner et al., 2016 on ASL; Tomasuolo et al., 2010 on LIS). As the SLCO learning environment is newly established and the size of the sample is quite small, more acquisition research with Deaf children from this environment is necessary in order to verify if it positively impacts their sign language acquisition.

At the theoretical level, this study attempts to apply Language Synthesis to account for the acquisition phenomena. The data confirm that Numeration from List 1 and Vocabulary Insertion are the two domains in which one may examine crosslingusitic interaction. This kind of research is still preliminary. In future, other structures which show typological differences or even similarities may be incorporated into the investigation.

## Author contributions

GT main and corresponding author, oversee the design, outline of the paper, theoretical framework, data collection and processing, as well as writing up and editing the paper. JL processing the data and writing up the results section of the paper.

### Conflict of interest statement

The authors declare that the research was conducted in the absence of any commercial or financial relationships that could be construed as a potential conflict of interest.

## Footnotes

^1^Emmorey et al. (2008) observe that bimodal bilinguals code blend much more often than code switch, indicating that lexical suppression is more costly than lexical selection. Further, given the articulatory constraints are removed, it is possible for bimodal bilinguals to actually produce both languages simultaneously.

^2^**Notation Conventions:** Following the conventions in the field, glosses for signs are capitalized (e.g., BOOK); glosses for a single sign are underscored (e.g., TAKE_A_PLANE); compound signs are marked with ^∧^ (e.g., WHAT_MONTH^∧^WHAT_DATE); nonmanuals and their scopes are marked by a line above the glosses; pointing signs are glossed as IX (e.g., IX-1 = first-person pronominal; IX_a_ refers to a locus in space); locations are indicated by subscripted letters “a, b, c,” whereas the subscripted letters “i, j, k” are used to mark coreferential meaning; the dashed line “-->--” indicates that the previous sign is held in space with one hand when the other hand continues signing. For classifier predicates in sign languages, the gloss begins with the verb root printed in small letters, to be followed by the classifier handshape that the referent stands for. For example, ‘be_located+CL_SASS_' means that the verb root is a locative and the classifier is represented by a size-and-shape specifier handshape. Additionally, the speech is transcribed by using the romanization system of the target language, such as *Jyutping* for Cantonese.

^3^While person and number features have been confirmed in many studies, there have been discussions about what formal morphosyntactic features are there with the verbal classifiers in sign languages. Zwitserlood (2003) offers a detailed discussion on her proposal of classifiers bearing gender features.

^4^See Talmy (2000) for the Figure and Ground relations in spoken languages.

^5^Localization may involve a pointing sign, role shift, a locative existential predicate, or simply directing a classifier to an R-locus in space without a downward movement. Further research is necessary to figure out their syntactic consequences.

^6^In Chinese, when verbs or prepositions select locations as their complements, it is necessary for the complement to take a localizer which denotes an axial part (Huang, 2009).

^7^A reviewer queried why the input before the SLCO Programme was not taken into consideration in this study. While these children joined a 45-minute sign language intervention programme weekly for at least one year before joining the SLCO Programme, they did not necessarily attend the sessions regularly because the Programme is not compulsory.

^8^A reviewer asked if the consistent difficulty in encoding the verb root ‘be_located' lends support to Koulidobrova's (2016) observation of object omission. In our data, objects were usually not omitted despite the absence of ‘be_located.' The DHs actually adopted an alternative strategy to encode the verb root (e.g. a lexical verb HAVE) having a similar meaning instead (see Table 7)

## References

[B1] Baker A. E. (2016). Incongruent grammar: can the model cope? Ling. Appr. Bilingualism 6, 760–762. 10.1075/lab.6.6.03bak

[B2] Benedicto E. Brentari D. (2004). Where did all the arguments go?: argument-changing properties of classifiers in ASL. Natl. Lang. Ling. Theor. 22, 743–810. 10.1007/s11049-003-4698-2

[B3] Branchini C. Donati C. (2016). Assessing lexicalism through bimodal eyes. Glossa J. General Ling. 1, 1–30. 10.5334/gjgl.29

[B4] Chen Pichler D. (2001). Word Order Variability and Acquisition in American Sign Language. Ph.D. dissertation, University of Connecticut.

[B5] Cogill-Koez D. (2000). Signed language classifier predicates: linguistic structures or schematic visual representation? Sign Lang. Ling. 3, 153–207. 10.1075/sll.3.2.03cog

[B6] Cormier K. Quinto-Pozos D. Sevcikova Z. Schembri A. (2012). Lexicalisation and de-lexicalisation processes in sign languages: comparing depicting constructions and viewpoint gestures. Lang. Commun. 32, 329–348. 10.1016/j.langcom.2012.09.004 23805017 PMC3688355

[B7] Crasborn O. Sloetjes H. (2008). Enhanced ELAN functionality for sign language corpora, in Proceedings of LREC 2008, Sixth International Conference on Language Resources and Evaluation, 39–43. Available online at: http://www.lrec-conf.org/proceedings/lrec2008

[B8] Donati C. Branchini C. (2013). Challenging linearization: simultaneous mixing in the production of bimodal bilinguals, in Challenges to Linearization, eds Biberauer T. Roberts I. (Berlin: Mouton De Gruyter), 93–128.

[B9] Emmorey K. Borinstein H. B. Thompson R. Gollan T. H. (2008). Bimodal bilingualism. Bilingual. Lang. Cogn. 11, 43–61. 10.1017/S1366728907003203 19079743 PMC2600850

[B10] Fung H.-M. C. Tang G. (2017). Code-blending of functional heads in Hong Kong Sign Language and Cantonese: a case study. Bilingual. Lang. Cogn. 19, 754–781. 10.1017/S1366728915000747

[B11] Glück S. Pfau R. (1998). On classifying classification as a class of inflection in German Sign Language, in Proceedings of ConSole VI, eds Cambier-Langeveld T. Lipták A. Redfor M. (Leiden: SOLE), 59–74.

[B12] Glück S. Pfau R. (1999). A Distributed Morphology account of verbal inflection in German Sign Language, in Proceedings of Console VII, eds Cambier-Langeveld T. Lipták A. Redford M. van der E. J. Torre (Leiden: SOLE), 65–80.

[B13] Harley H. (2014). On the identity of roots. Theor. Ling. 40, 1–53. 10.1515/tl-2014-0010

[B14] Henner J. Caldwell-Harris C. L. Novogrodsky R. Hoffmeister R. (2016). American Sign Language syntax and analogical reasoning skills are influenced by early acquisition and age of entry to signing schools for the Deaf. Front. Psychol. 7:1982. 10.3389/fpsyg.2016.01982 28082932 PMC5183573

[B15] Huang C. T.-J. (1990). On ‘be' and ‘have' in Chinese. Bull. Inst. Hist. Philol. Acad. Sin. 59, 43–64.

[B16] Huang C. T.-J. (2009). Lexical decomposition, silent categories, and the Localizer Phrase. Yuyanxue Luncong 39, 86–122.

[B17] Hulk A. Müller N. (2000). Bilingual first language acquisition at the interface between syntax and pragmatics. Bilingual. Lang. Cogn. 3, 227–244. 10.1017/S1366728900000353

[B18] Koulidobrova H. (2012). When the Quiet Surfaces: ‘Transfer’ of Argument Omission in the Speech of ASL-English Bilinguals. Ph.D. dissertation, University of Connecticut.

[B19] Koulidobrova H. (2016). Language interaction effects in bimodal bilingualism: argument omission in the languages of hearing ASL-English bilinguals. Ling. Appr. Bilingual. 75, 583–613. 10.1075/lab.13047.kou

[B20] Lam W. S. (2009). Early Phrase Structure in Hong Kong Sign Language: A Case Study. Ph.D. dissertation, The Chinese University of Hong Kong.

[B21] Lillo-Martin D. (1999). Modality effects and modularity in language acquisition: the acquisition of American Sign Language, in Handbook of Language Acquisition, eds Ritchie W. Bhatia T. (San Diego, CA: Academic Press), 531–567.

[B22] Lillo-Martin D. Berk S. (2003). Acquisition of constituent order under delayed language exposure, in Proceedings from 27^*th*^ Annual Boston University Conference on Language Development, eds Beachley B. Brown A. Conlin F. (Somerville, MA: Cascadilla Press), 484–495.

[B23] Lillo-Martin D. de Quadros R. M. Chen Pichler D. Fieldsteel Z. (2014). Language choice in bimodal bilingual development. Front. Psychol. 5:1163. 10.3389/fpsyg.2014.01163 25368591 PMC4202712

[B24] Lillo-Martin D. de Quadros R. M. Koulidobrova H. Chen Pichler D. (2010). Bimodal bilingual cross-language influence in unexpected domains, in Language Acquisition and Development: Proceedings of GALA 2009, eds Costa J. Castro A. Lobo M. Pratas F. (Newcastle upon Tyne: Cambridge Scholars Press), 264–275.

[B25] Lillo-Martin D. Koulidobrova H. de Quadros R. M. Chen Pichler D. (2012). Bilingual language synthesis: evidence from wh-questions in bimodal bilinguals, in Proceedings of the 36th Annual Boston University Conference on Language Development, eds Biller A. K. Chung E. Y. Kimball A. E. (Somerville, MA: Cascadilla Press), 302–314.

[B26] Lillo-Martin D. de Quadros R. M. Chen Pichler D. (2016). The development of bimodal bilingualism: implications for linguistics theory. Ling. Appr. Bilingual. 6, 719–755. 10.1075/lab.6.6.01lil PMC5461974 28603576

[B27] Lindert R. (2001). Hearing Families with Deaf Children: Linguistic and Communicative Aspects of American Sign Language Development. Ph.D. dissertation, University of California, Berkeley.

[B28] MacSwan J. (2000). The architecture of the bilingual language faculty: evidence from intrasentential code switching. Bilingual. Lang. Cogn. 3, 37–54. 10.1017/S1366728900000122

[B29] MacSwan J. (2005). Codeswitching and generative grammar: a critique of the MLF model and some remarks on “modified minimalism.” Bilingual. Lang. Cogn. 8, 1–21. 10.1017/S1366728904002068

[B30] Meisel J. M. (2011). First and Second Language Acquisition: Parallels and Differences. Cambridge: Cambridge University Press.

[B31] Newport E. Meier R. (1985). The acquisition of American Sign Language, in The Crosslinguistic Study of Language Acquisition, ed Slobin D. (Mahwah, NJ: Lawrence Erlbaum), 881–938.

[B32] Ng H. Y. I. (2014). The Construction and Validation of the Cantonese Spoken Word Recognition Test (CanSWORT) to Measure Word Recognition Ability of Cantonese-speaking Population. Ph.D. dissertation, The Chinese University of Hong Kong.

[B33] Palmer J. L. (2015). Bimodal Bilingual Word Order Acquisition. Ph.D. dissertation, Gallaudet University.

[B34] Petitto L. A. Katerelos M. Levy B. G. Gauna K. Tétreault K. Ferraro V. (2001). Bilingual signed and spoken language acquisition from birth: implications for the mechanisms underlying early bilingual language acquisition. J. Child Lang. 28, 453–496. 10.1017/S0305000901004718 11449947

[B35] Pfau R. Aboh E. (2012). On the syntax of spatial adpositions in sign languages. MIT Work. Papers Ling. 65, 83–104.

[B36] Schembrei A. (2003). Rethinking ‘classifiers’ in signed languages, in Perspectives on Classifier Constructions in Sign Languages, ed Emmorey K. (Mahwah, NJ: Psychology Press), 3–34.

[B37] Schick B. (1990). The effects of morphosyntactic structure on the acquisition of classifier predicates in ASL, in Sign language research: Theoretical issues, ed Lucas C. (Washington, DC: Gallaudet University Press), 358–374.

[B38] Supalla T. (1982). Structure and Acquisition of Verbs of Motion and Location in American Sign Language. Ph.D. dissertation, University of California, San Diego, CA.

[B39] Supalla T. (1986). Classifier system in American Sign Language, in Noun Classes and Categorization, ed Craig C. (Amsterdam/Philadelphia: John Benjamins Publishing Company), 181–214.

[B40] Sze F. (2000). Word order of Hong Kong Sign Language, in Cross-linguistic Perspectives in Sign Language Research, eds Baker A. van den Bogaerde B. Crasborn O. (Hamburg: Signum), 163–192.

[B41] T'sou B. Lee T. Tung P. Chan A. Man Y. To C. (2006). Hong Kong Cantonese Oral Language Assessment Scale. Hong Kong: City University of Hong Kong Press.

[B42] Talmy L. (2000). Toward a cognitive semantics. Vol. 1: Concept Structuring Systems. Cambridge, Mass: MIT Press.

[B43] Tang G. Lam S. Yiu K.-M. C. (2014). Language development of deaf children in a sign bilingual and co-enrollment environment, in Bilingualism and Bilingual Deaf Education, eds Marschark M. Tang G. Knoors H. (New York, NY: Oxford University Press), 313–341.

[B44] Tang G. Sze F. Lam S. (2007).” Acquisition of simultaneous constructions by deaf children of Hong Kong Sign Language,” in Simultaneity in Signed Languages: Form and Function, eds Vermeerbergen M. Leeson L. Crasborn O. (Amsterdam: Benjamins), 283–316.

[B45] Tang G. Yiu C. Lam S. (2015). Awareness of Hong Kong sign language and manually coded chinese by deaf students, in Educating Deaf Learners: Creating a Global Evidence Base, eds Knoors H. Marschark M. (New York, NY: Oxford University Press), 117–148.

[B46] Tomasuolo E. Fellini L. Renzo A. D. Volterra V. (2010). Assessing lexical production in deaf signing children with the Boston Naming Test. Lang. Interact. Acquisit. 1, 110–128. 10.1075/lia.1.1.07tom

[B47] van den Bogaerde B. Baker A. (2005). Code mixing in mother-child interaction in deaf families. Sign Lang. Linguist., 8, 153–176. 10.1075/sll.8.1.08bog

[B48] Yuen K. C. P. Ng I. H. Y. Luk B. P. K. Chan S. K. W. Chan S. C. S. Kwok I. C. L.. (2008). The development of Cantonese Lexical Neighborhood Test: a pilot study. Int. J. Pediatric Otorhinolaryngol. 72, 1121–1129. 10.1016/j.ijporl.2008.03.025 18485493

[B49] Zwitserlood I. (2003). Classifying Hand Configurations in Nederlandse Gebarentaal (Sign Language of the Netherlands). Ph.D. dissertation, Utrecht University.

[B50] Zwitserlood I. (2008). Morphology below the level of the sign–frozen forms and classifier predicates, in Signs of the Time: Selected Papers from TISLR 2004, ed J. Quer (Hamburg: Signum), 251–272.

