# Corrigendum: Acquisition of Classifier Constructions in HKSL by Bimodal Bilingual Deaf Children of Hearing Parents

**DOI:** 10.3389/fpsyg.2018.01637

**Published:** 2018-09-19

**Authors:** Gladys W. L. Tang, Jia Li

**Affiliations:** Department of Linguistics and Modern Languages, Centre for Sign Linguistics and Deaf Studies, The Chinese University of Hong Kong, Shatin, Hong Kong

**Keywords:** bimodal bilingualism, word order, classifier constructions, language acquisition, HKSL, Cantonese, deaf children, coenrollment

In the original article, we neglected to include the funder **CUHK-Faculty of Arts**, **The Publication Subvention Fund to Gladys W. L. Tang**. The authors apologize for this error and state that this does not change the scientific conclusions of the article in any way.

The original article has been updated.

## Conflict of interest statement

The authors declare that the research was conducted in the absence of any commercial or financial relationships that could be construed as a potential conflict of interest.

